# Are mutagenic non D-loop direct repeat motifs in mitochondrial DNA under a negative selection pressure?

**DOI:** 10.1093/nar/gkv299

**Published:** 2015-04-08

**Authors:** Lakshmi Narayanan Lakshmanan, Jan Gruber, Barry Halliwell, Rudiyanto Gunawan

**Affiliations:** 1 Institute for Chemical and Bioengineering, ETH Zurich, Zurich 8093, Switzerland; 2 Department of Chemical and Biomolecular Engineering, National University of Singapore, Singapore 117585, Singapore; 3 Yale-NUS College, Department of Biochemistry, Neurobiology and Ageing Program, National University of Singapore, Singapore 117599, Singapore; 4 Department of Biochemistry, Neurobiology and Ageing Program, Centre for Life Sciences (CeLS), National University of Singapore, Singapore 117599, Singapore; 5 Swiss Institute of Bioinformatics, Quartier Sorge - Batiment Genopode, 1015 Lausanne, Switzerland

## Abstract

Non D-loop direct repeats (DRs) in mitochondrial DNA (mtDNA) have been commonly implicated in the mutagenesis of mtDNA deletions associated with neuromuscular disease and ageing. Further, these DRs have been hypothesized to put a constraint on the lifespan of mammals and are under a negative selection pressure. Using a compendium of 294 mammalian mtDNA, we re-examined the relationship between species lifespan and the mutagenicity of such DRs. Contradicting the prevailing hypotheses, we found no significant evidence that long-lived mammals possess fewer mutagenic DRs than short-lived mammals. By comparing DR counts in human mtDNA with those in selectively randomized sequences, we also showed that the number of DRs in human mtDNA is primarily determined by global mtDNA properties, such as the bias in synonymous codon usage (SCU) and nucleotide composition. We found that SCU bias in mtDNA positively correlates with DR counts, where repeated usage of a subset of codons leads to more frequent DR occurrences. While bias in SCU and nucleotide composition has been attributed to nucleotide mutational bias, mammalian mtDNA still exhibit higher SCU bias and DR counts than expected from such mutational bias, suggesting a lack of negative selection against non D-loop DRs.

## INTRODUCTION

Mutations in mitochondrial DNA (mtDNA) have been associated with mitochondrial dysfunction, diseases and the ageing process (1,2). In particular, the accumulation of mtDNA deletions in cells plays a causal role in age-related skeletal muscle fiber atrophy in sarcopenia and neuronal loss in the substantia nigra of aged individuals and Parkinson's disease patients (3,4). These deletions typically involve the loss of coding-genes in mtDNA outside the D-loop region. While the mechanism of deletion mutagenesis is not precisely known, direct repeat (DR) motifs have been frequently observed flanking the breakpoints of mtDNA deletions in different organisms including human (5–8). Our previous study showed that mtDNA deletion breakpoints in human and rhesus monkey are significantly nearer to DRs than what would be expected by random chance (9). The DR motifs have also been suggested to act as pre-existing hotspots for mtDNA rearrangements (10). Commonly held hypotheses on mtDNA deletion mutagenesis further assert a causal relationship, in which DRs mediate mtDNA misalignments during mtDNA replication or recombination, leading to the formation of mtDNA deletions (11,12).

The origin and evolution of DR motifs in mtDNA are more poorly understood than those in nuclear DNA (nDNA). Sequence repeats in nDNA have been investigated more extensively than those in mtDNA due to their involvement in neurodegenerative disorders as well as their practical applications such as in DNA fingerprinting (13–15). DNA repeat sequences can be categorized based on the distance between the repeats (tandem or distantly located), the length of the repeats (short or long), or the genome location (non-coding regions or coding regions). Different types of repeat sequences are believed to evolve by different mechanisms. For example, tandem repeats (TRs), i.e. repeats that appear in a series, have been proposed to arise by point mutations. The number of TRs can expand or shrink within genomes by a replication slippage mechanism (16,17). Meanwhile, repeat motifs in non-coding genome regions are hypothesized to evolve without any selection pressure, whereas repeats within coding regions are expected to be under selection due to the need to prevent frame shift mutations (14,18). Past studies on repeat motifs in mtDNA had mainly focused on TRs in the regulatory D-loop region (19–23). A large variation of TR sizes has been reported in the D-loop, from 2 bp to several 100s bp (22). These TRs have been hypothesized to occur by slipped strand mis-pairing, recombination, transposition or competitive strand displacement (23–25). The presence of D-loop TRs has further been suggested to evolve under selection pressures based on protein binding sequences (19,23) and secondary structure formation (stem–loop) (21,26).

The evolution of non D-loop DRs has not been studied as extensively as TRs in D-loop regions, and the key factors that determine the number of these mutagenic DRs are still poorly understood. These DRs are typically small in size (<20 bp) and non-tandem in nature (9,27,28). Meanwhile, DRs in D-loop have not been implicated in mtDNA deletions associated with neuromuscular diseases and ageing. One possible explanation is that the resulting mutant molecule may have partially or completely missing heavy-strand replication origin (O_H_). This and other mutations affecting the sequence of mtDNA replication origins, could render the mutant molecules at a replicative disadvantage with respect to wild-type mtDNA (29,30). Consequently, the corresponding mutant mtDNA would not be able to accumulate to high levels necessary to cause mitochondrial dysfunction. While there is a well-known deletion breakpoint hotspot at one of the ends of D-loop region (e.g. 16 071 bp in human), the corresponding deletions are not flanked by DRs (31,32). Many DR-independent hypotheses have been proposed to explain the mutagenesis at this hotspot, involving stem–loop structures (33), non-homologous recombination of the triple stranded region of D-loop (31,34), and defects in nuclear encoded polymerase gamma and twinkle helicase (32,35). For these reasons and as in other related studies (10,27,36), we therefore focus our investigation on DRs found outside the mtDNA D-loop region.

Because of the mutagenicity of DRs and the association between mtDNA deletions and the ageing process, the number of non D-loop DRs in mtDNA sequences has previously been linked to the longevity of the organisms (10,27). A study using a compendium of 61 mammalian mtDNA reported that long-lived mammals possess fewer long DRs (length ≥ 12 bp) in their mtDNA. As longer DRs are expected to hybridize more readily and are thus considered to be more mutagenic than shorter DRs, the above observation led to the hypothesis that the number of long DRs in mtDNA imposes a constraint on mammalian lifespan (27). Following this initial observation, a second study reported a negative correlation between total DR mutagenicity score (TMS) of mtDNA (calculated using the number of DRs ≥ 10 bp) and species lifespan among 65 mammals, again suggesting that natural selection in long-lived mammals favored fewer long DRs in mtDNA (10). Our own earlier study on mtDNA deletions in four mammals: human, rhesus monkey, mouse and rat, also supported a possible negative selection pressure against the number of long DRs (≥12 bp) in the mtDNA of human and rhesus monkey, but not mouse and rat (9). However, a recent analysis of 529 mtDNA sequences, including 202 mammalian mtDNA, showed only a weak negative correlation between DR mutagenicity score and lifespan among mammals, which became insignificant after contrast analysis, demonstrating the importance of correcting for phylogenetic relatedness among the species in such comparative analysis (36).

Interestingly, despite their mutagenic properties, DRs are more readily found in mtDNA. The frequency of DRs in DNA sequences has been shown to increase with bias in nucleotide composition (14). However earlier studies, including our own, have shown that random DNA sequences of the same length and base composition as native mtDNA have lower DR counts (9,27), indicating the involvement of other factors. We hypothesized therefore that the number of DRs might be affected not only by global features of mtDNA sequences (e.g. length, bias and skew of base composition, synonymous codon usage (SCU)), but also by the specific ordering of nucleotides and codons within individual genes. However, how much each of these mtDNA sequence features influences the number of DRs is not immediately obvious. Knowledge about these factors can provide insights into the evolutionary forces affecting DR counts, which would aid in establishing the existence and nature of evolutionary selection on DR motifs in mtDNA. In this study, we gathered a compendium of mtDNA sequences, lifespan and body mass data of 294 mammals in order to examine (i) the link between the organism longevity and long DRs in mtDNA, and (ii) the degrees to which different features of mtDNA sequence influence the occurrence of DRs.

## MATERIALS AND METHODS

### Mitochondrial DNA sequences, lifespan and body mass dataset

We compiled annotated mitochondrial DNA sequences within the taxonomic class of mammalia (*n =* 294) from the NCBI database. We then grouped the mtDNA sequences according to their taxonomic orders, including only mammalian orders with at least 10 species with known lifespan and body mass. The lifespan was taken as the maximum reported value across three different sources: the animal ageing and longevity (AnAge) database (37), animal diversity web database (http://animaldiversity.org) and longevity records (38). Meanwhile, the body mass was averaged over the values reported across the three aforementioned databases. In addition, we obtained 236 mtDNA sequences within the taxonomic class of aves from the NCBI database. The complete list of animals used in this study can be found in Supplementary Data.

### Analysis of DR count and DR mutagenicity score

The number of DRs in a mtDNA sequence was determined by exhaustively scanning the sequence for all possible DR pairs of lengths >4 bp (see Supplementary Data). As done in previous studies (27,36) and for reasons mentioned above, the regulatory D-loop region of the mtDNA was omitted in the counting of DRs. The TMS of mtDNA sequences was calculated using two different methods based on DR-mediated deletion frequencies in engineered bacteriophage λ DNA (10,39) and in yeast mtDNA (36,40).

### Generation of random DNA sequences

In order to investigate the relative influence of global and gene specific sequence properties of mtDNA on the DR count, we generated several types of selectively randomized sequences based on human mtDNA by:

- reshuffling gene positions in mtDNA (randomized gene order, RGO);
- reshuffling nucleotides within rRNA-coding genes;
- reshuffling nucleotides within tRNA-coding genes;
- reshuffling nucleotides within protein-coding genes;
- reshuffling nucleotides within the mtDNA sequence, excluding D-loop region (Full);
- reshuffling codons within protein-coding genes;
- randomizing synonymous codon usage in protein-coding genes (unbiased synonymous codon usage, USCU);
- randomizing nucleotide usage (NU), i.e. random DNA sequences with the same length as human mtDNA but using uniform composition of the four nucleotide bases.

In the generation of USCU random sequences, nucleotide sequences and codon start positions for protein-coding genes were obtained from mtDNA annotation. Each codon was first translated into the corresponding amino acid (AA) based on the vertebrate mtDNA genetic code (from NCBI annotation). For validation and for identification of rare abnormalities in the initiation and stop codons, the translated AA sequences were then compared with the reference sequences available in NCBI database. Subsequently, we converted the validated AA sequences back to nucleotide sequences in which synonymous codons were used with equal frequency, thereby neutralizing any bias on codon usage. In addition, we generated Full and USCU random sequences for the other mammalian mtDNA in our compendium in the same manner as described above.

### Analysis of synonymous codon usage bias and strand asymmetry

The nucleotide sequences of protein-coding genes in each mtDNA in mammalian and avian datasets were first concatenated, omitting the start and stop codons for the calculation of SCU bias. The synonymous codon usage bias was measured by computing the effective number of codons (*N*_c_) using the CodonW package (http://codonw.sourceforge.net). Meanwhile, strand asymmetry in mtDNA sequences was represented using GC-skew ((%G − %C)/(%G + %C)) and AT-skew ((%A − %T)/(%A + %T)) (41).

### Phylogenetic analysis

Amino acid sequences of 13 mtDNA encoded proteins in the mammalian and avian mtDNA compendiums were first concatenated and then used for the phylogenetic analysis. Sequence alignments were performed using ClustalW (42) and MUSCLE (43). Based on the sequence alignments from ClustalW and MUSCLE, phylogenetic relationships among the species in the dataset were constructed and phylogenetically independent contrast analysis was subsequently performed using PHYLIP (http://evolution.genetics.washington.edu/phylip.html; *proml* and *contrast* functions, respectively).

### Statistical analysis

Correlations and statistical analyses were performed using built-in subroutines in MATLAB (Version R2012b; MathWorks, Inc.). Two sample *t*-test and Mann–Whitney U-test (MWU) were employed for comparisons of mean and median values of DR counts between short- and long-lived mammalian subpopulations, respectively. In addition, two sample Kolmogorov–Smirnov (K–S) test was used to assess whether DR counts from short- and long-lived subpopulations come from the same distribution. Meanwhile, Z-test was used for the comparison of DR counts between mtDNA and different randomized sequences. Unless stated otherwise, statistical significance corresponded to a *P*-value ≤0.05.

## RESULTS

### DR counts in mammalian mtDNA

**Figure 1.**
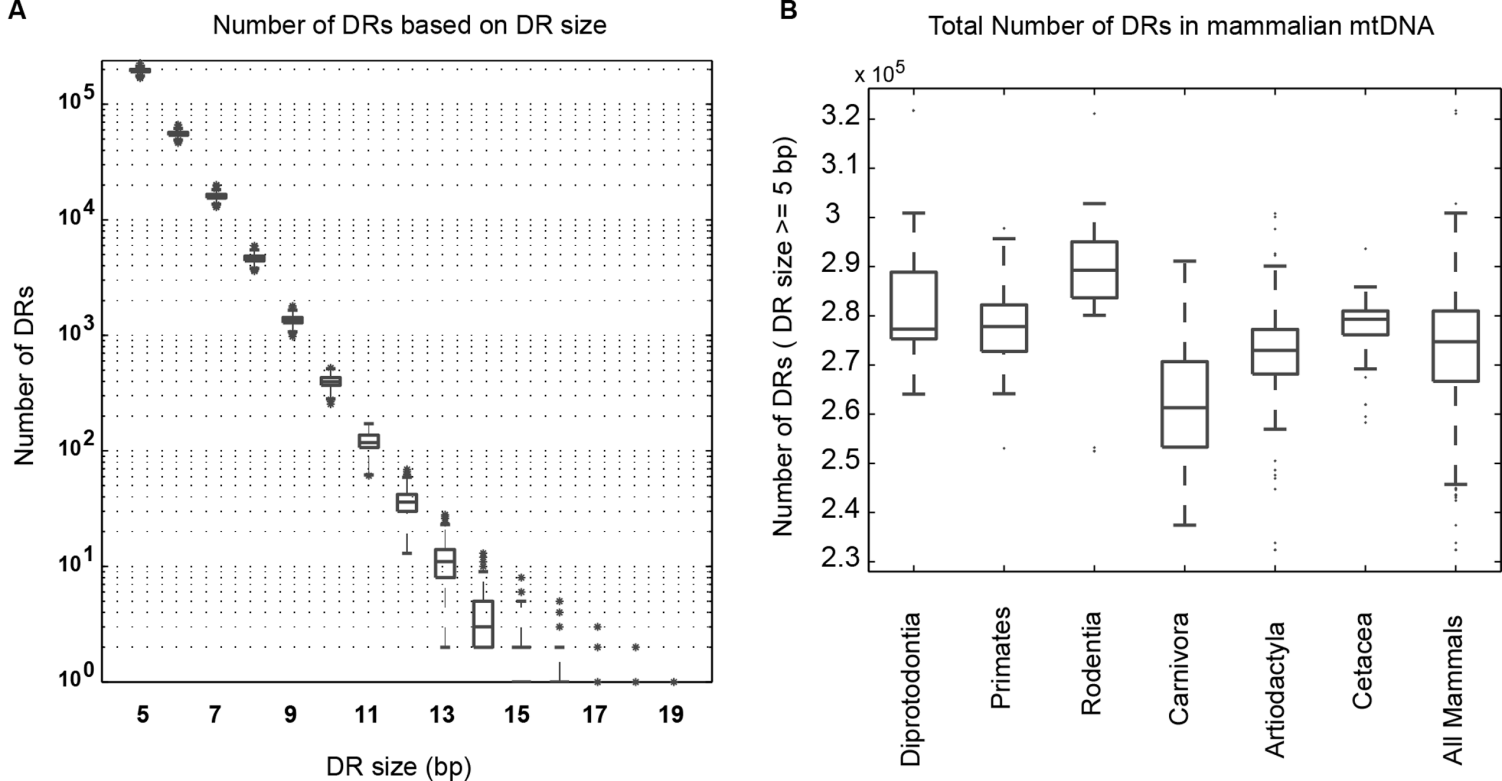
Number of DR motifs in mammalian mtDNA sequences. (**A**) Distribution of DR counts for each DR size in 294 mammalian mtDNA sequences. The number of DRs decreases exponentially with increasing DR size. The longest DR in the dataset is 19 bp. (**B**) Comparison of total DR count (i.e. for all DRs of length ≥ 5 bp) among mammals in different taxonomic orders. Each boxplot shows the median and spread of total numbers of DRs within each taxonomic order.

Our compendium consists of mtDNA sequences from 294 mammals within six taxonomic orders, namely: Diprotodontia (*n* = 12), Primates (*n* = 50), Rodentia (*n* = 19), Carnivora (*n* = 61), Artiodactyla (*n* = 117) and Cetacea (*n* = 35). Mammalian mtDNA sequences have similar lengths of around 16.5 kb. Furthermore, the gene content in mtDNA among mammals is highly conserved, consisting of two ribosomal RNAs, 22 transfer RNAs and 13 proteins (44). For each mtDNA sequence, we determined the number of DR motifs of different sizes from 5 bp. Direct repeats of length ≤4 bp were not considered in this study, because the majority of such DRs possessed a positive free energy of hybridization and in principle, could not spontaneously hybridize (9). The DR counts intuitively decreased with larger DR size in an exponential manner (Figure 1A). Pairwise comparisons of median DR counts among taxonomic orders indicated significant differences for DR sizes from 5 to 12 bp, with a few exceptions primarily involving Diprotodontia (two-sided Mann–Whitney U (MWU) test; Supplementary Table S1). However, DRs ≥13 bp are infrequent, the number of which did not vary considerably across taxonomic orders other than Carnivora. As shown in Figure 1B, the mtDNA sequences within Carnivora typically possessed fewer total DRs (size ≥ 5 bp) than those in other taxonomic orders for unknown reasons (one-sided MWU-test, *P* ≤ 0.01, Supplementary Table S2).

### Long DRs and longevity of mammals

We re-examined the prevailing hypothesis that the number of potentially highly mutagenic long DRs (≥13 bp) constrains the longevity of organisms (27), and that such motifs are thus subjected to a negative selection during mtDNA evolution (10). For this purpose, we first examined the correlation between the logarithm of species lifespan and mtDNA DR count. However, body mass has been suggested as a major confounding factor when inferring correlations between lifespan and other parameters of interest (45). Indeed, the lifespan and body mass of mammals in our dataset are strongly correlated (*ρ* = 0.68; *P* < 10^−4^). In addition, the mtDNA of extant mammals share an evolutionary relationship with respect to a common ancestor. Thus, it is important to correct for phylogenetic relatedness in analyzing the correlation between lifespan and DR count for example by using contrast analysis (45,46). For these reasons, in the following, we first calculated the residuals of DR count and logarithm of lifespan by controlling for the logarithm of body mass. Subsequently, we performed a contrast analysis on the above residuals to correct for phylogenetic relatedness among the species. Finally, we evaluated the correlation between phylogenetically independent contrasts (PICs) of the residual (log-) lifespan and DR count. In addition to DR counts, we also analyzed the correlation between the PICs of residual (log-) lifespan and (log-) TMS, controlling for (log-) body mass (10,36).

We did not find any significant correlation between the PICs of residual lifespan and DR count in our mammalian dataset for long DRs ≥ 13 bp (*ρ* = –0.11, *P*-value = 0.07; ClustalW-Phylip), as well as for other DR sizes (see Supplementary Table S3). The results from ClustalW and MUSCLE sequence alignments were practically identical (MUSCLE-Phylip: *ρ* = −0.10, *P*-value = 0.08; Supplementary Table S3). From the analyses of mammals within each taxonomic order, we found a significant negative correlation between lifespan and long DRs (≥13 bp) only in Cetacea (see Supplementary Table S4). Upon reanalyzing the mammalian data by excluding Cetacea, we observed a comparatively less significant correlation between lifespan and the number of long DRs ≥ 13 bp (ClustalW-Phylip: *ρ* = –0.07, *P*-value = 0.24; MUSCLE-Phylip: *ρ* = –0.07, *P*-value = 0.24).

We applied the above partial correlation analysis to investigate the relationship between lifespan and TMS defined in previous studies (10,36). In agreement with our findings above, we did not find any consistent and significant correlations between the PICs of (log-) lifespan and (log-) TMS among all mammals in our compendium, as well as among those within individual taxonomic orders (see Supplementary Table S5). The lack of correlation between lifespan and TMS above contradicted the negative correlation reported previously for 65 mammals in the study by Khaidakov *et al*. (see Figure 1 in (10)). However, by reanalyzing the raw data of Khaidakov *et al*., we found that the previously observed negative correlation became insignificant after correcting for phylogenetic relatedness among the species (ClustalW-Phylip: *ρ* = 0.003, *P*-value = 0.98; MUSCLE-Phylip: *ρ* = 0.003, *P*-value = 0.98). This finding is consistent with an observation in a recent study, where contrast analysis also abrogated the correlation between lifespan and DR mutagenicity score (36). Therefore, there appears to be no consistent and significant relationship between the organism lifespan and the mutagenicity of DRs in the mtDNA, measured either by the number of long DRs or by TMS.

**Figure 2.**
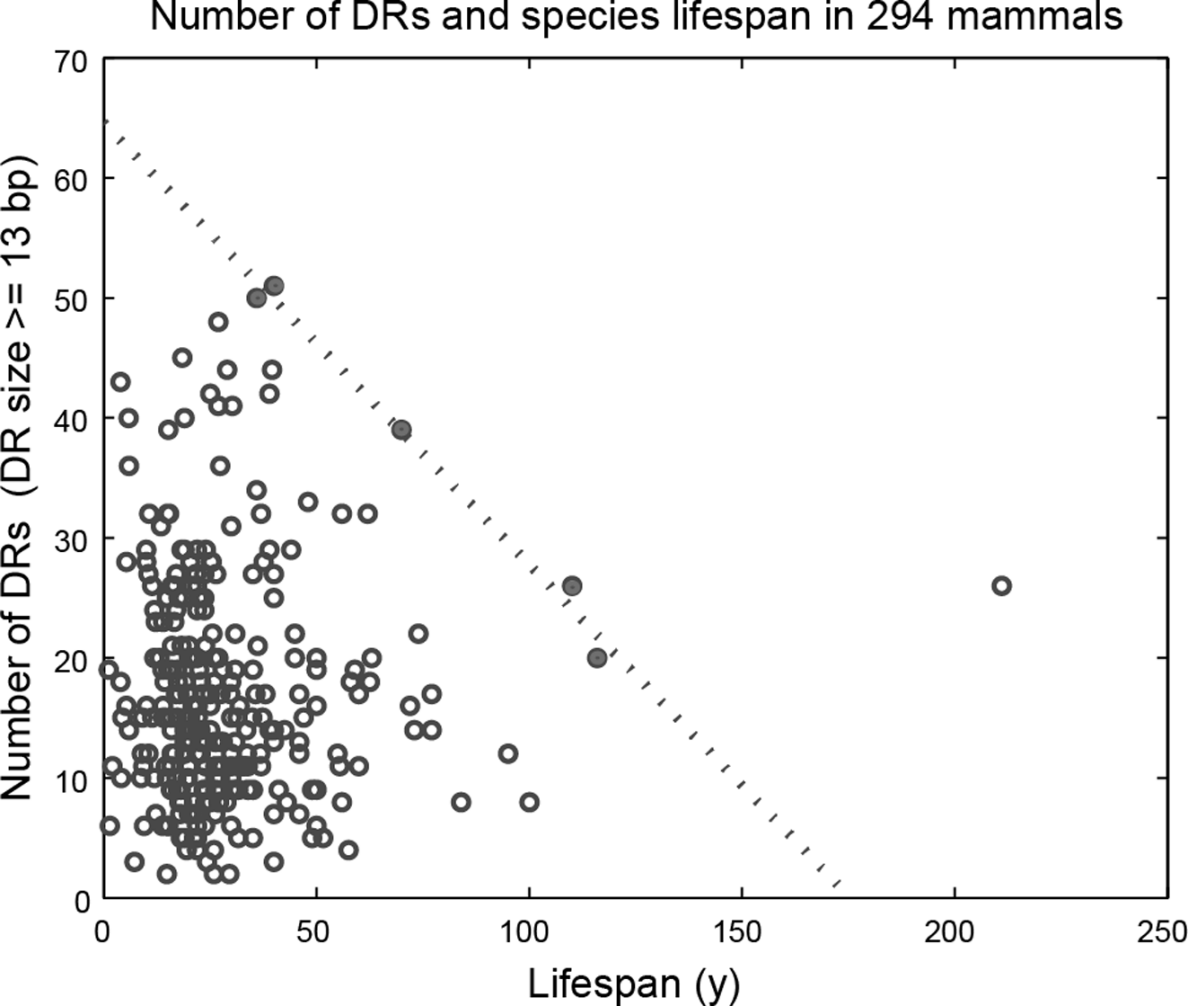
Relationship between species lifespan and DR counts. The species lifespan and the number of long DRs (≥13 bp) in 294 mammals. The linear upper constraint line was drawn based on the least-square fitting using five species (filled circles). The data point for the bowhead whale (lifespan = 211 years) was not included in the constraint line calculation.

The weak correlation between lifespan and the number of DRs was also reported in the dataset used in one of the earlier studies (27). However, in the plot of the number of DRs ≥13 bp versus the species lifespan (see Figure 1 in (27)), long-lived mammals visually appeared to possess fewer long DRs in their mtDNA in comparison to short-lived animals. As shown in Figure 2, we observed a similar trend for our mammalian dataset. In a previous study, a constraint line was subsequently drawn to represent an upper limit of long DR counts as a function of the organism longevity (27). Based on this constraint relationship, the author stated that long-lived mammals have fewer long DRs (≥13 bp) than short-lived mammals and that the numbers of long DR in short-lived mammals are randomly distributed below the constraint line (27). Here, we were concerned that such a constraint line and hypotheses might sensitively depend on the small sample size of organisms with lifespan >50 years. Therefore, in the following analysis, we divided the mammals in the dataset into two subpopulations: short-lived and long-lived, according to whether their lifespans were lower or higher than a threshold, respectively.

We first analyzed the difference in the mean and median counts of long DRs between the two subpopulations for different thresholds, specifically the 50th, 75th and 90th percentiles of the lifespan data. In this case, we found no significant difference in the mean and median numbers of long DRs ≥13 bp regardless of the threshold (two-sided *t*-test and two-sided MWU-test; Supplementary Tables S6 and S7). Interestingly, for moderately sized DRs between 7 and 12 bp, the mean and median DR counts of the short-lived subpopulations were consistently lower than those of the long-lived subpopulations (using thresholds of 75th and 90th percentile; one-sided t-test and one-sided MWU-test, *P* < 0.05), which is opposite to the hypothesized trend. We repeated the above analysis for mammals within specific taxonomic orders with a sample size *n* ≥ 50, i.e. Primates, Carnivora and Artiodactyla. Again, the mean and median number of long DRs (≥13 bp) in short- and long-lived subpopulation did not differ from each other (Supplementary Tables S6 and S7), except for the taxonomic order Artiodactyla when using 50th percentile threshold.

Furthermore, we compared the DR counts of the short-lived subpopulation with those of the long-lived subpopulation using two sample K–S test, with the null hypothesis that the two samples come from the same distribution. In this analysis, we could not reject the null hypothesis for DR size ≥13 bp among all mammals, as well as among those in individual taxonomic orders (*P*-value ≥ 0.07; see Supplementary Table S8). The K–S tests for other DR sizes (5–12 bp and ≥ 5 bp) among all mammals led to rejections of the null hypothesis when using 75th and 90th percentile thresholds (*P*-value ≤ 0.03; Supplementary Table S8). However, the differences between the short- and long-lived subpopulations here arose mostly due to large lifespan variations across different taxonomic orders, as suggested by the lack of statistical significance from K–S tests for the same DR sizes within individual taxa (see Supplementary Table S8). Therefore, in contradiction with the prevailing hypotheses, we did not find any evidence supporting a negative correlation nor a constraint relationship between lifespan and long DR counts among the mammals in our compendium.

### Determinants of DR counts in mtDNA

Our earlier results and also findings from another study have shown that human mtDNA possessed a significantly higher DR count than random DNA sequences of the same length and nucleotide composition (9,27). We have previously postulated that this observation could be due to the occurrence of recurrent motifs in coding genes associated with the structure of proteins and RNAs. However, different global attributes of mtDNA sequences, such as sequence lengths, gene arrangements, codon usage and base compositions (bias and skew), as well as gene specific attributes such as structural motifs within protein- and RNA-coding genes, can affect the number of DRs in mtDNA. The relative importance of such mtDNA attributes in influencing the number of DRs in mtDNA is not obvious.

**Figure 3.**
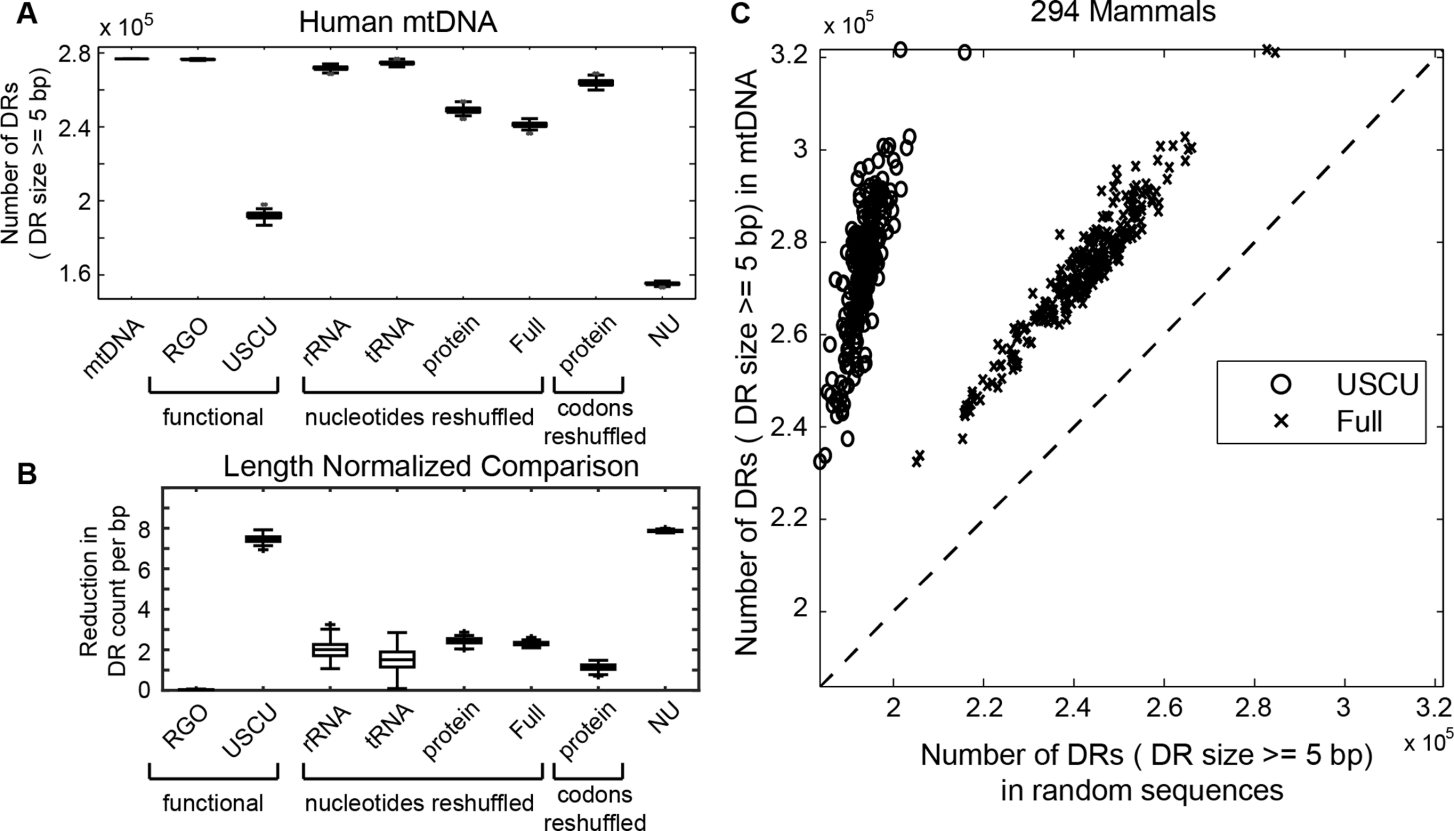
DR counts in random DNA sequences. (**A**) Comparison of total DR counts in human mtDNA and eight different selectively randomized sequences. (**B**) Normalized reduction in DR counts (count/bp) in randomized sequences in comparison to human mtDNA. (**C**) Comparison of total DR counts between native mtDNA and selectively randomized sequences USCU and Full in 294 mammals.

In order to assess how much each of the aforementioned attributes affects the DR count in human mtDNA, we generated eight selectively randomized sequences (see ‘Materials and Method’ section). Each set of the selectively randomized sequences differs from human mtDNA in one of the attributes mentioned above. Figure 3A gives the comparison of total DR counts (≥5 bp) in these selectively randomized sequences and native human mtDNA (also see Supplementary Table S9). We found that reshuffling the order of genes (in RGO sequences) did not lead to a significant difference in the total DR count of human mtDNA sequence (two-sided Z-test; *P*-value = 0.11; also see Supplementary Table S10). However, each of the remaining randomization techniques resulted in a lower total DR count than that seen in the native human mtDNA (one-sided Z-test; *P* < 10^−4^; also see Supplementary Table S11).

For comparison purposes, Figure 3B gives the relative reductions of total DR counts (≥5 bp), normalized with respect to the sequence length involved in the randomization (for individual DR sizes, see Supplementary Table S12). Randomizing nucleotide sequences in coding genes, including those for rRNA, tRNA and protein, led to similar relative reductions in total DR count per bp, as shown Figure 3B. The most significant reductions resulted from USCU (unbiased SCU) and NU (unbiased nucleotide usage) random sequences. These observations also apply for small to moderately sized DRs (5–10 bp). Interestingly the counts of large DRs (≥11 bp) were little changed with randomization (see Supplementary Table S12). The above results thus indicated that fully functional mtDNA sequences with USCU and sequences with uniform nucleotide frequencies have much fewer DRs than native human mtDNA.

The large difference in the DR counts between NU random sequences and human mtDNA was perhaps not surprising, as NU sequences shared only one attribute with human mtDNA which was the length. In generating NU sequences we neutralized, among other things, the bias and skew in nucleotide frequencies, which have been known to increase DR counts in DNA sequences (14). In contrast, reshuffling only the nucleotides in human mtDNA without changing their composition (see random sequences ‘Full’ in Figure 3B) led to much smaller reductions in DR count, confirming the importance of nucleotide composition on DR occurrence. Nevertheless, as mentioned earlier, human mtDNA still possesses more DRs than sequences with the same nucleotide composition (9,27). By comparing the total DR count of each mammalian mtDNA in our dataset with the average total DR counts (≥5 bp) of the corresponding ‘Full’ random sequences in Figure 3C, we showed that the above observation applies generally to mammals.

The USCU random sequences, unlike the rest of selectively randomized sequences considered in this study, maintained functional amino acid (AA) sequence information in the protein-coding genes, i.e. these selectively random sequences would produce fully functional proteins. The strong effect of SCU bias on DR counts was particularly interesting, considering that most synonymous codons differed only by one nucleotide in the last position. In order to understand better the relationship between the SCU bias and the occurrence of DRs, we generated USCU random sequences for each of the mtDNA in our mammalian dataset and repeated the analysis above. Figure 3C shows the comparison of the average total DR counts (≥5 bp) of USCU random sequences (*n* = 100 for each species) and the total DR count of each mtDNA sequence in the compendium. The comparison demonstrated that on average, USCU random sequences harbored significantly fewer DRs than the native mtDNA molecules among all mammals in this study. In other words, DR counts in these mammals could be dramatically reduced if codon usage bias was relaxed. Moreover, this means that, without changing the AA sequences of protein coding genes, DRs could be diminished through silent single nucleotide mutations.

**Figure 4.**
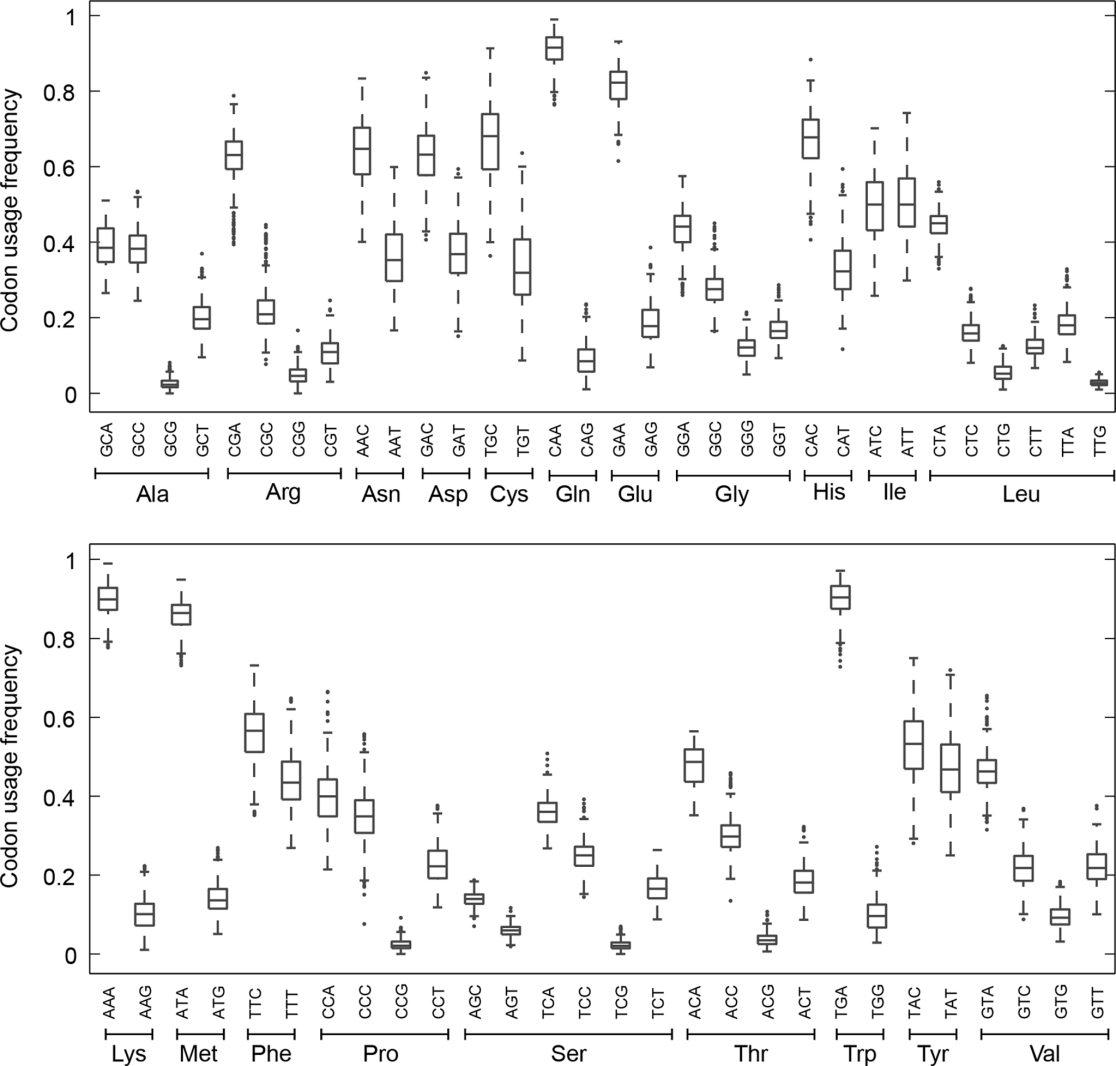
Synonymous codon usage in mammalian mtDNA sequences. The boxplots show the distributions of codon usage frequencies among 294 mammalian mtDNA for different amino acids. Synonymous codons were grouped together according to the amino acids they encode.

Because of the strong influence of SCU bias on the number of DRs in human mtDNA, we also examined the SCU bias in each mammalian mtDNA sequence in the dataset, by analyzing concatenated protein-coding sequences (see ‘Materials and Methods’ section). We observed a significant bias in the SCU for all amino acids, except for isoleucine and tyrosine (Figure 4). The SCU bias showed two general trends, namely: (i) codons containing G in the third position were used the least, and (ii) for amino acids with four synonymous codons (i.e. Ala, Arg, Gly, Pro, Thr and Val), codons with A in the third position were used the most, followed by C, T and G, in the order of frequency of use. The above observation on codon usage frequency is in good agreement with previously published results for mammalian mtDNA, but our study includes a substantially larger dataset than the previous studies (44,47,48).

We further investigated the relationship between the DR counts and the extent of SCU bias, measured using the effective number of codons (*N*_c_) (see ‘Materials and Methods’ section) (49). The metric *N*_c_ was chosen based on a recommendation from a survey of existing metrics for SCU bias, especially for comparing homologous genes across different species (50). The values of *N*_c_ can span between 20 and 61, where a lower value indicates a higher SCU bias. The *N*_c_ values for concatenated protein encoding sequences among mtDNA in the mammalian compendium ranged between 38.4 and 48.7 with a median value of 43.0, indicating a moderate SCU bias. The extent of SCU bias varied among mammalian taxonomic orders (two-sided MWU-test; *P* ≤ 0.03; see Supplementary Figure S1). The *N*_c_ of 42.64 for human mtDNA in our analysis is in agreement with a previously reported value of 42.6 (48). While the SCU bias varies among individual protein-coding genes, a previous analysis of 9862 human mtDNA sequences also reported a moderate codon usage bias with *N*_c_ values between 31 and 41 (51).

**Figure 5.**
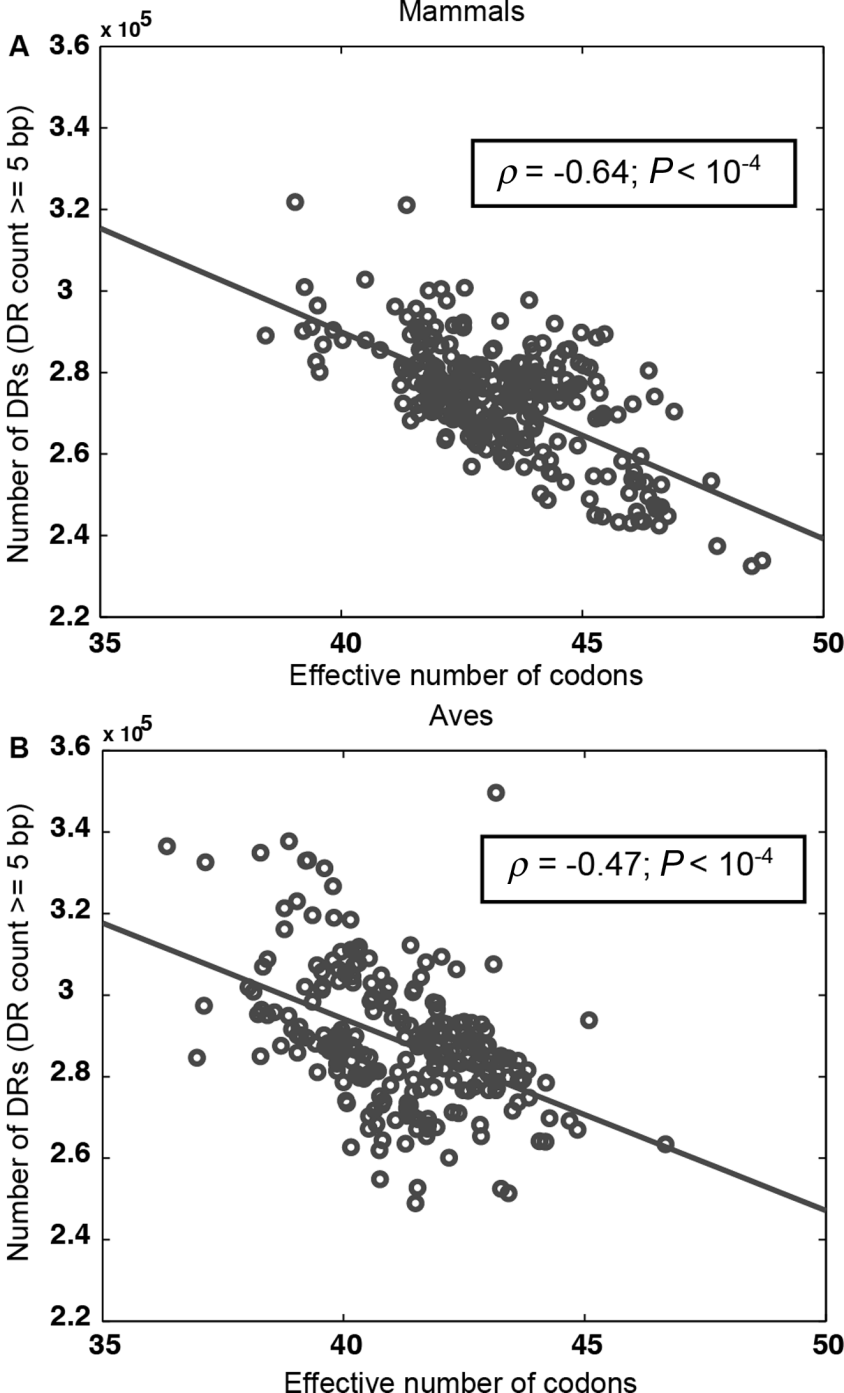
Correlations between SCU bias and DR counts in mammalian and avian mtDNA. The effective number of codons and total mtDNA DR count in (**A**) 294 mammals and (**B**) 236 aves. The linear correlation coefficient (*ρ*) and the *P*-value were shown on each plot.

Synonymous codons primarily differ in the nucleotide at the third codon position. Hence, there exists a co-dependency between bias in nucleotide composition and that in SCU. In analyzing the correlation between *N*_c_ values and DR counts below, we employed a partial correlation analysis, controlling for GC and AT skew as well as GC content in mtDNA. In addition, we employed contrast analysis to correct for phylogenetic relatedness. We observed a negative correlation between the *N*_c_ values and the total DR counts (≥5 bp) without any control, as shown in Figure 5A (*ρ* = −0.64, *P*-value < 10^−4^; for individual taxonomic orders see Supplementary Figure S2). In other words, the number of DRs in mtDNA increased with the extent of SCU bias. The correlation was lower but still statistically significant after controlling for nucleotide usage bias, strand asymmetry and phylogenetic relatedness (ClustalW-Phylip: *ρ* = −0.32, *P*-value < 10^−4^; Supplementary Table S13). A similar finding was also observed for different taxonomic orders in the mammalian compendium, except for those with sample sizes ≤35 (namely Diprotodontia, Rodentia and Cetacea). The results from ClustalW and MUSCLE were in good agreement with each other (see Supplementary Tables S13 and S14). Repeating the analysis for a compendium of 236 mtDNA sequences from the taxonomic class aves, we again found a statistically significant correlation between the extent of SCU bias and the total DR counts after corrections for bias and skew in nucleotide composition and phylogenetic relatedness (see Figure 5B, before control: *ρ* = −0.47, *P*-value < 10^−4^; after control using ClustalW: *ρ* = −0.15, *P*-value = 0.02; Supplementary Tables S13 and S14). For individual DR sizes in mammals and aves, the correlations generally became weaker with increasing DR lengths (see Supplementary Tables S13 and S14).

## DISCUSSION

### Do long DRs constrain the longevity of mammals?

In revisiting the prevailing hypotheses that the number of mutagenic long DRs in mtDNA constrains the longevity of animals (27) and that such motifs are under a negative selection (10), we could not find any significant supporting evidence from our analysis of 294 mammalian mtDNA. In particular, we did not observe any significant correlation between the mutagenicity of long DRs and lifespan after controlling for body mass and phylogenetic relatedness. In addition, we did not find any differences in the distributions of the numbers of long DRs (≥13 bp) between short- and long-lived subpopulations in the mammalian dataset. On the contrary, for a few moderately sized DRs (7–12 bp), we found the opposite trend of what was expected from the hypothesis, where the short-lived subpopulation had lower mean and median DR counts than the long-lived subpopulation. Therefore, the results of our first analysis suggested that any selective pressure on long DRs, if it existed, is not strongly associated with mammalian longevity, and that the number of DRs does not appear to impose any limit on the lifespan. Nevertheless, the hypothesized relationship may still be valid in certain mammalian lineages, as suggested by the significant negative correlation between lifespan and long DR count in Cetacea. Such an exception is perhaps not surprising as cetaceans live in a different habitat from other mammals, and are hypothesized to have undergone distinct adaptations in their metabolism (52) including mitochondrial membrane proteins (53).

### Determinants of DR counts in mtDNA

Our analysis of human mtDNA showed that the native mtDNA contained more DRs than any of the selectively randomized sequences considered in this study. We found that global sequence properties, particularly nucleotide composition and SCU bias, are strong determinants of DRs in human mtDNA. While bias and skew in nucleotide frequencies are known to increase the occurrence of DRs in DNA sequences (14), to the best of our knowledge, the involvement of SCU bias has not been previously reported. However, since bias in nucleotide frequencies and SCU are related to each other, the importance of SCU bias could result from an indirect effect through changes in nucleotide composition.

By performing partial correlation analysis, controlling for nucleotide composition (GC and AT skew and GC content), we confirmed the importance of SCU bias in mammalian mtDNA within our dataset. Specifically, we found that the numbers of DRs among mammalian mtDNA increased with the extent of SCU bias even when corrected for nucleotide composition and phylogenetic relatedness among the species. We also observed a significant, albeit weaker, correlation between the SCU bias and DR count in avian mtDNA, indicating that our finding was not limited to mammals. The higher DR count with the greater bias in the SCU is explained by the fact that protein-coding genes made up a majority of mammalian mtDNA sequences and that recurring use of a subset of codons in these genes would increase the chance of forming repeat sequences. The results above thus provided novel evidence for the causal effect of the SCU bias on the formation of DRs in mammalian mtDNA.

### Origin and evolution of DRs in mammalian mtDNA sequences

Bias and skew of base composition in mtDNA have been proposed to arise due to nucleotide mutational bias during mtDNA replication (41). Nucleotide mutational bias describes the non-uniform frequency of point mutations where specific types of point mutations occur more frequently than others (54). In mammalian mtDNA, point mutations predominantly involve nucleotides C and T. This bias has been attributed to the directional and context-dependent mutagenesis process (where mutagenesis of a nucleotide is affected by its neighboring nucleotides) during mtDNA replication, transcription and repair (41,55–57). Meanwhile, a number of evolutionary factors have been used to explain bias in SCU in DNA sequences, such as translational efficiency, accuracy of protein synthesis, tRNA abundance, gene expression levels and nucleotide mutational bias (54,58). Studies on the evolution of SCU in mtDNA genes have suggested that the SCU bias in mtDNA was also associated with nucleotide mutational bias (47,48,57).

**Figure 6.**
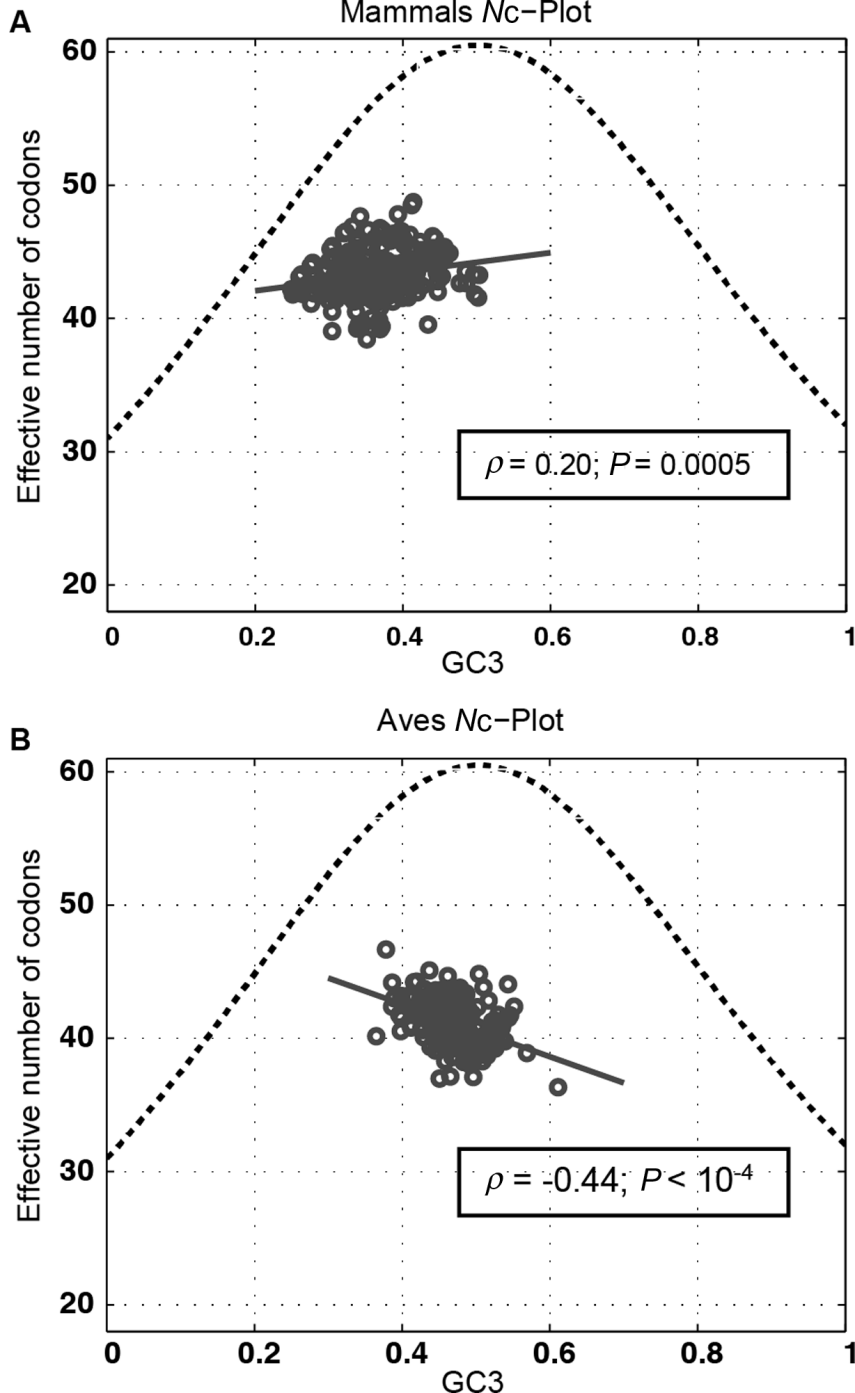
*N*_c_-plot. Relationship between the expected SCU bias due to only nucleotide mutational bias (*N*_c_*; dotted line), the SCU bias (*N*_c_; circles) and the GC fractions at third codon positions in (**A**) 294 mammalian mtDNA sequences and (**B**) 236 avian mtDNA sequences. The linear correlation coefficient (*ρ*) and the *P*-value were shown on each plot for the correlation between the effective number of codons and the GC3.

Considering that uneven usage of nucleotides and synonymous codons could cause increased occurrences of DRs in mtDNA, a higher extent in each of these factors will lead to more DRs, thereby enhancing the propensity of DR-mediated mtDNA misalignments and deletion mutagenesis. However, as shown in Figure 3C, mammalian mtDNA possess higher DR counts in the non D-loop region than random sequences with the same bias and skew in nucleotide frequencies, which contradicts the expected trend if mutagenic DRs are under a negative selection. Similarly, if such a negative selection exists, then the extent of SCU bias in mtDNA should be lower than what would be expected due to only uneven nucleotide composition that resulted from nucleotide mutational bias. In the following, we calculated the expected value of bias in SCU (*N*_c_*) as a function of the GC fraction at the third codon position (GC3) (49). A significant deviation of *N*_c_ from *N*_c_* indicates a possible selection pressure on the SCU bias (49). Figure 6 shows the comparison of *N*_c_* and *N*_c_ for mammalian and avian mtDNA. The *N*_c_ values of mammals and aves were lower than *N*_c_*, implying that the extents of SCU bias were higher than expected from nucleotide mutational bias. Thus, evolution appears to favor a higher SCU bias in spite of the resulting increased occurrences of mutagenic DR motifs.

Furthermore, the *N*_c_ values of mammalian mtDNA displayed a weak positive correlation with the GC3 compositions (*ρ_xy_* = 0.20; *P*-value = 0.0005), while the avian *N*_c_ were negatively correlated with the GC3 compositions (*ρ_xy_* = −0.44; *P* < 10^−4^). We also observed taxonomic order specific differences in the correlation between *N*_c_ and GC3 (Supplementary Figure S3). Diprotodontia, Rodentia, Artiodactyla and Cetacea did not exhibit any significant correlation between *N*_c_ with GC3 (*P* ≥ 0.08), whereas Primates exhibited a negative correlation (*ρ_xy_* = −0.44; *P*-value = 0.0013) and Carnivora showed a positive correlation (*ρ_xy_* = 0.33; *P*-value = 0.01). Importantly, the correlations between SCU bias and GC3 above deviated significantly from the expected SCU bias dependence on GC3. These results support the existence of additional factors influencing the SCU bias other than nucleotide mutational bias. In the analysis of SCU bias among individual protein coding genes in human mtDNA, Levin *et al*. observed a lack of correlation between *N*_c_ and GC3. In this study, the authors suggested that the SCU bias in human mtDNA arises as an adaptation towards the usage of translation efficient codons (51).

Taken together, in contrast to previous suggestions our results reveal a lack of negative selection pressure against DR motifs in mtDNA, even of long-lived species. Given their likely role in mtDNA deletion formation, this may at first seem unexpected. However this result can be understood in the context of evolutionary theories of aging, based on Peter Medawar's insight that the force of natural selection declines rapidly after sexual maturity. This is the case because genotypes that have detrimental phenotypes only late in life will adversely affect survival only after the relevant genes have been passed on to the next generation. Therefore, deleterious genotypes or mutations whose effects are confined to old age evade evolutionary selection (59). While higher counts of DRs may increase the occurrences of mtDNA misalignment and deletion mutations, such mutations need to first accumulate to a significant fraction (≥60%) of the mtDNA population in a cell before affecting cellular energetics (60). This expansion of mutant mtDNA molecules in somatic cells is a time-consuming process (61–64), and could take years to decades. Consequently, their deleterious effects are only observable in aged tissues, and the diseases associated with mtDNA deletions, such as sarcopenia and some neurodegenerative diseases, only become symptomatic late in life, typically well after sexual maturity. For these reasons, it may not be surprising that the number of DRs in mtDNA does not appear to be under strong evolutionary selection.

## SUPPLEMENTARY DATA

Supplementary Data are available at NAR Online.

SUPPLEMENTARY DATA
