# Supplementary material for: Are mutagenic non D-loop direct repeat motifs in mitochondrial DNA under a negative selection pressure?
Source: Nucleic Acids Res. 2015 Apr 8;43(8):4098–108. doi: 10.1093/nar/gkv299 (PMC4417187; doi:10.1093/nar/gkv299)
Supplement: SUPPLEMENTARY DATA [file supp_43_8_4098__index.html]

Are mutagenic non D-loop direct repeat motifs in mitochondrial DNA under a negative selection pressure? — Are mutagenic non D-loop direct repeat motifs in mitochondrial DNA under a negative selection pressure? — SUPPLEMENTARY DATA 

# Are mutagenic non D-loop direct repeat motifs in mitochondrial DNA under a negative selection pressure?

## SUPPLEMENTARY DATA

**Files in this Data Supplement:**

- SUPPLEMENTARY DATA
- SUPPLEMENTARY DATA
- SUPPLEMENTARY DATA
