# Supplementary material for: Are mutagenic non D-loop direct repeat motifs in mitochondrial DNA under a negative selection pressure?
Source: Nucleic Acids Res. 2015 Apr 8;43(8):4098–108. doi: 10.1093/nar/gkv299 (PMC4417187; doi:10.1093/nar/gkv299)
Supplement: SUPPLEMENTARY DATA [file supp_gkv299_nar-02864-z-2014-File008.pdf]

Supplementary Tables

Table of Contents

Supplementary Table S1: Inter-taxonomic order comparison of DR counts. 3

Supplementary Table S2: Comparison of DR counts in Carnivora with other taxonomic orders. 4

Supplementary Table S3: Correlation analysis between phylogenetically independent contrasts (PICs) of residual lifespan and residual DR counts after controlling for body mass in 294 mammals. 5

Supplementary Table S4: Correlation analysis between phylogenetically independent contrasts (PICs) of residual lifespan and residual DR counts after controlling for body mass in taxonomic orders within mammals. 6

Supplementary Table S5: Correlation analysis between phylogenetically independent contrasts (PICs) of residual lifespan and residual DR total mutagenicity scores (TMS) after controlling for body mass. 7

Supplementary Table S6: Comparison of Median DR counts between short lived and long lived mammals 8

Supplementary Table S7: Comparison of Mean DR counts between short lived and long lived mammals 12

Supplementary Table S8: Comparison of DR count distributions between short-lived and long-lived mammals 16

Supplementary Table S9: Percent reduction in DR counts between human mtDNA and randomized DNA sequences 17

Supplementary Table S10: Two-sided comparison of DR counts between human mtDNA and randomized DNA sequences 18

Supplementary Table S11: One-sided comparison of DR counts between human mtDNA and randomized DNA sequences 19

Supplementary Table S12: Normalized Reduction in DR counts between human mtDNA and randomized DNA sequences. **20**

Supplementary Table S13: Correlation between PICs of residual N_c and residual DR counts using ClustalW-Phylip **21**

Supplementary Table S14: Correlation between PICs of residual N_c and residual DR counts using MUSCLE-Phylip **22**

Supplementary Table S1: Inter-taxonomic order comparison of DR counts.

Two-sided MWU-test was used to test the null hypothesis that DR counts in the compared pair of datasets arise from continuous distributions with equal median values, against the alternative that the medians are unequal. Pairwise comparisons with p-values ≤ 0.05 are highlighted in red.

**Datasets Compared**		**Two-sided MWU-test p-values**									
**Dataset 1**	**Dataset 2**	**≥ 5 bp**	**5 bp**	**6 bp**	**7 bp**	**8 bp**	**9 bp**	**10 bp**	**11 bp**	**12 bp**	**≥ 13 bp**
Diprotodontia	Primates	0.55	0.44	0.53	0.71	0.81	0.84	0.98	0.41	0.06	0.04
Diprotodontia	Rodentia	0.09	0.10	0.09	0.08	0.05	0.04	0.04	0.01	0.01	0.06
Diprotodontia	Carnivora	0.00	0.00	0.00	0.00	0.00	0.00	0.01	0.03	0.57	0.84
Diprotodontia	Artiodactyla	0.00	0.00	0.00	0.01	0.10	0.22	0.32	0.92	0.13	0.10
Diprotodontia	Cetacea	0.87	0.60	0.89	0.63	0.24	0.16	0.08	0.02	0.02	0.12
Primates	Rodentia	0.00	0.00	0.00	0.00	0.01	0.00	0.00	0.01	0.10	0.93
Primates	Carnivora	0.00	0.00	0.00	0.00	0.00	0.00	0.00	0.00	0.00	0.00
Primates	Artiodactyla	0.00	0.00	0.00	0.00	0.00	0.06	0.05	0.16	0.20	0.10
Primates	Cetacea	0.95	0.80	0.96	0.36	0.23	0.01	0.01	0.02	0.41	0.32
Rodentia	Carnivora	0.00	0.00	0.00	0.00	0.00	0.00	0.00	0.00	0.00	0.02
Rodentia	Artiodactyla	0.00	0.00	0.00	0.00	0.00	0.00	0.00	0.00	0.01	0.22
Rodentia	Cetacea	0.00	0.00	0.00	0.00	0.01	0.05	0.05	0.07	0.14	0.33
Carnivora	Artiodactyla	0.00	0.00	0.00	0.00	0.00	0.00	0.00	0.00	0.00	0.01
Carnivora	Cetacea	0.00	0.00	0.00	0.00	0.00	0.00	0.00	0.00	0.00	0.03
Artiodactyla	Cetacea	0.00	0.00	0.00	0.00	0.00	0.00	0.00	0.00	0.05	0.82

Supplementary Table S2: Comparison of DR counts in Carnivora with other taxonomic orders.

One-sided MWU-test was used to test the null hypothesis that DR counts in the compared pair of datasets arise from continuous distributions with equal median values, against the alternative that Carnivora exhibit a lower median DR count. Pairwise comparisons with p-values ≤ 0.05 are highlighted in red.

Datasets Compared		One-sided MWU-test p-values									
		DR lengths									
**Dataset 1**	**Dataset 2**	**5**	**6**	**7**	**8**	**9**	**10**	**11**	**12**	**≥ 13**	**≥ 5**
**Carnivora**	**Diprotodontia**	0.00	0.00	0.00	0.00	0.00	0.01	0.01	0.29	0.42	0.00
**Carnivora**	**Primates**	0.00	0.00	0.00	0.00	0.00	0.00	0.00	0.00	0.00	0.00
**Carnivora**	**Rodentia**	0.00	0.00	0.00	0.00	0.00	0.00	0.00	0.00	0.01	0.00
**Carnivora**	**Artiodactyla**	0.00	0.00	0.00	0.00	0.00	0.00	0.00	0.00	0.00	0.00
**Carnivora**	**Cetacea**	0.00	0.00	0.00	0.00	0.00	0.00	0.00	0.00	0.01	0.00

Supplementary Table S3: Correlation analysis between phylogenetically independent contrasts (PICs) of residual lifespan and residual DR counts after controlling for body mass in 294 mammals.

Sequence alignments required for phylogenetic tree construction were generated separately using ClustalW and MUSCLE. Phylogenetic tree and the PICs were obtained using Phylip.

DR size	Correlation between PICs of residual LS and residual DR counts			
	ClustalW-Phylip		MUSCLE-Phylip	
	ρ	p-value	ρ	p-value
**5 bp**	-0.09	0.14	-0.09	0.12
**6 bp**	-0.07	0.27	-0.07	0.25
**7 bp**	-0.03	0.59	-0.03	0.58
**8 bp**	-0.01	0.91	-0.01	0.92
**9 bp**	0.04	0.53	0.04	0.49
**10 bp**	0.03	0.61	0.04	0.53
**11 bp**	-0.01	0.92	0.00	0.97
**12 bp**	-0.03	0.65	-0.02	0.70
**≥ 13 bp**	-0.11	0.07	-0.10	0.08
**≥ 5 bp**	-0.07	0.21	-0.08	0.19

Supplementary Table S4: Correlation analysis between phylogenetically independent contrasts (PICs) of residual lifespan and residual DR counts after controlling for body mass in taxonomic orders within mammals.

Sequence alignments required for phylogenetic tree construction were generated separately using ClustalW and MUSCLE. Phylogenetic tree and the PICs were obtained using Phylip. Correlations with p-values ≤ 0.05 are highlighted in red.

DR size	Correlation between PICs of residual lifespan and residual DR counts											
	Diprotodontia				Primates				Rodentia			
	ClustalW-Phylip		MUSCLE-Phylip		ClustalW-Phylip		MUSCLE-Phylip		ClustalW-Phylip		MUSCLE-Phylip	
	ρ	p-value	ρ	p-value	ρ	p-value	ρ	p-value	ρ	p-value	ρ	p-value
**5 bp**	0.24	0.48	0.29	0.38	-0.01	0.93	-0.02	0.90	-0.07	0.79	-0.23	0.36
**6 bp**	0.23	0.49	0.29	0.38	0.01	0.93	0.02	0.90	-0.01	0.96	-0.17	0.50
**7 bp**	0.24	0.47	0.31	0.36	0.03	0.82	0.04	0.80	0.00	0.99	-0.13	0.61
**8 bp**	0.28	0.40	0.35	0.28	0.06	0.68	0.06	0.68	0.07	0.80	-0.07	0.78
**9 bp**	0.37	0.26	0.44	0.17	0.21	0.14	0.21	0.14	0.22	0.37	0.03	0.90
**10 bp**	0.42	0.19	0.48	0.14	0.33	0.02	0.35	0.01	0.41	0.09	0.09	0.72
**11 bp**	0.30	0.37	0.35	0.29	0.26	0.07	0.31	0.03	0.25	0.32	-0.06	0.83
**12 bp**	0.28	0.40	0.35	0.30	0.13	0.39	0.17	0.25	0.17	0.51	-0.10	0.70
**≥ 13 bp**	0.46	0.15	0.61	0.05	-0.13	0.39	-0.12	0.43	0.04	0.88	-0.13	0.61
**≥ 5 bp**	0.24	0.47	0.30	0.37	0.01	0.97	0.00	0.98	-0.04	0.87	-0.20	0.42

DR size	Correlation between PICs of residual lifespan and residual DR counts											
	Carnivora				Artiodactyla				Cetacea			
	ClustalW-Phylip		MUSCLE-Phylip		ClustalW-Phylip		MUSCLE-Phylip		ClustalW-Phylip		MUSCLE-Phylip	
	ρ	p-value	ρ	p-value	ρ	p-value	ρ	p-value	ρ	p-value	ρ	p-value
**5 bp**	-0.24	0.06	-0.24	0.06	-0.14	0.14	-0.13	0.16	0.16	0.36	0.15	0.40
**6 bp**	-0.22	0.09	-0.22	0.10	-0.13	0.16	-0.12	0.19	0.22	0.21	0.21	0.22
**7 bp**	-0.13	0.31	-0.13	0.31	-0.10	0.27	-0.09	0.33	0.23	0.19	0.23	0.19
**8 bp**	-0.05	0.69	-0.06	0.68	-0.10	0.30	-0.08	0.40	0.23	0.19	0.24	0.18
**9 bp**	0.02	0.91	0.01	0.93	-0.09	0.36	-0.07	0.48	0.22	0.21	0.23	0.19
**10 bp**	-0.01	0.95	-0.01	0.93	-0.11	0.24	-0.09	0.34	0.14	0.44	0.14	0.44
**11 bp**	0.01	0.94	0.01	0.94	-0.09	0.32	-0.07	0.42	-0.09	0.60	-0.10	0.59
**12 bp**	0.04	0.77	0.05	0.71	0.00	0.99	0.01	0.89	-0.41	0.02	-0.41	0.02
**≥ 13 bp**	-0.18	0.16	-0.18	0.16	-0.06	0.51	-0.04	0.66	-0.39	0.02	-0.39	0.02
**≥ 5 bp**	-0.22	0.09	-0.22	0.09	-0.13	0.15	-0.13	0.18	0.18	0.30	0.18	0.32

Supplementary Table S5: Correlation analysis between phylogenetically independent contrasts (PICs) of residual lifespan and residual DR total mutagenicity scores (TMS) after controlling for body mass.

Sequence alignments required for phylogenetic tree construction were generated separately using ClustalW and MUSCLE. Phylogenetic tree and the PICs were obtained using Phylip. TMS values were calculated for long DRs (≥ 10 bp) using the methods proposed in two earlier articles (Khaidakov et al., (a) and Yang et al., (b)). In accordance with the earlier studies, logarithm to the base 10 was used for log transformation of lifespan and TMS values in Khaidakov et.al. method and logarithm to the base 2 was used for log transformation in Yang et.al., method. Correlations with p-values ≤ 0.05 are highlighted in red.

Taxonomic group	Khaidakov et al., method				Yang et al., method			
	ClustalW-Phylib		MUSCLE-Phylip		ClustalW-Phylib		MUSCLE-Phylip	
	ρ	p-value	ρ	p-value	ρ	p-value	ρ	p-value
**All Mammals**	-0.08	0.19	-0.08	0.18	-0.06	0.29	-0.01	0.81
**Diprotodontia**	0.03	0.92	0.08	0.81	0.41	0.21	0.13	0.71
**Primates**	-0.31	0.03	-0.32	0.03	0.05	0.74	0.04	0.76
**Rodentia**	0.17	0.49	-0.08	0.76	0.22	0.38	-0.11	0.66
**Carnivora**	0.13	0.33	0.13	0.31	-0.09	0.48	-0.14	0.28
**Artiodactyla**	-0.14	0.13	-0.13	0.17	-0.10	0.28	0.02	0.84
**Cetacea**	0.17	0.34	0.19	0.28	-0.23	0.19	-0.11	0.55

(a) Khaidakov, M., Siegel, E.R. and Shmookler Reis, R.J. (2006) Direct repeats in mitochondrial DNA and mammalian lifespan. *Mech Ageing Dev*, **127**, 808-812.

(b) Yang, J.-N., Seluanov, A. and Gorbunova, V. (2013) Mitochondrial inverted repeats strongly correlate with lifespan: mtDNA inversions and aging. *PLoS One*, **8**, e73318.

Supplementary Table S6: Comparison of Median DR counts between short lived and long lived mammals

MWU-test was used to test the null hypothesis that DR counts in short-lived and long-lived datasets arise from continuous distributions with equal median values, against the following alternative hypotheses (a) median DR count values are unequal, (b) median DR count value in short-lived dataset is higher than the long-lived dataset and (c) median DR count value in short-lived dataset is lower than the long-lived dataset. Pairwise comparisons with p-values ≤ 0.05 are highlighted in red.

Percentile	DR size	short-lived ≠ long lived	short-lived > long lived	short-lived < long lived
		p-value	p-value	p-value
**Mammals**				
50	**5**	0.84	0.58	0.42
	**6**	0.54	0.73	0.27
	**7**	0.27	0.86	0.14
	**8**	0.18	0.91	0.09
	**9**	0.24	0.88	0.12
	**10**	0.12	0.94	0.06
	**11**	0.39	0.8	0.2
	**12**	0.61	0.69	0.31
	**≥ 13**	0.58	0.29	0.71
	**≥ 5**	0.69	0.66	0.34
75	**5**	0.17	0.92	0.08
	**6**	0.13	0.94	0.06
	**7**	0.04	0.98	0.02
	**8**	0.01	0.99	0.01
	**9**	0.01	1	0
	**10**	0	1	0
	**11**	0	1	0
	**12**	0	1	0
	**≥ 13**	0.45	0.77	0.23
	**≥ 5**	0.12	0.94	0.06

**90**	**5**	0.08	0.96	0.04
	**6**	0.03	0.98	0.02
	**7**	0	1	0
	**8**	0	1	0
	**9**	0	1	0
	**10**	0	1	0
	**11**	0	1	0
	**12**	0.04	0.98	0.02
	**≥ 13**	0.88	0.56	0.44
	**≥ 5**	0.04	0.98	0.02
**Primates[a]**		**short-lived ≠ long lived**	**short-lived > long lived**	**short-lived < long lived**
**50**	**5**	0.17	0.92	0.08
	**6**	0.19	0.91	0.1
	**7**	0.11	0.95	0.05
	**8**	0.08	0.96	0.04
	**9**	0.04	0.98	0.02
	**10**	0.01	1	0
	**11**	0.02	0.99	0.01
	**12**	0.03	0.99	0.01
	**≥ 13**	0.49	0.76	0.25
	**≥ 5**	0.15	0.93	0.08
**75**	**5**	0.77	0.62	0.39
	**6**	0.57	0.72	0.28
	**7**	0.38	0.82	0.19
	**8**	0.28	0.87	0.14
	**9**	0.19	0.91	0.09
	**10**	0.12	0.94	0.06
	**11**	0.35	0.83	0.17
	**12**	0.81	0.6	0.4
	**≥ 13**	0.23	0.12	0.89
	**≥ 5**	0.63	0.69	0.31

Carnivora[a]		short-lived ≠ long lived	short-lived > long lived	short-lived < long lived
50	**5**	0.27	0.87	0.13
	**6**	0.21	0.9	0.1
	**7**	0.1	0.95	0.05
	**8**	0.04	0.98	0.02
	**9**	0.04	0.98	0.02
	**10**	0.01	0.99	0.01
	**11**	0.05	0.98	0.02
	**12**	0.2	0.9	0.1
	**≥ 13**	0.48	0.24	0.76
	**≥ 5**	0.23	0.89	0.11
75	**5**	0.81	0.6	0.4
	**6**	0.69	0.66	0.35
	**7**	0.38	0.81	0.19
	**8**	0.21	0.9	0.11
	**9**	0.18	0.91	0.09
	**10**	0.09	0.96	0.05
	**11**	0.37	0.82	0.19
	**12**	0.66	0.67	0.33
	**≥ 13**	0.74	0.37	0.64
	**≥ 5**	0.69	0.66	0.35
Artiodactyla		short-lived ≠ long lived	short-lived > long lived	short-lived < long lived
50	**5**	0.03	0.01	0.99
	**6**	0.03	0.01	0.99
	**7**	0.06	0.03	0.97
	**8**	0.08	0.04	0.96
	**9**	0.19	0.09	0.91
	**10**	0.08	0.04	0.96
	**11**	0.02	0.01	0.99
	**12**	0.05	0.03	0.97
	**≥ 13**	0.01	0.01	0.99

	**≥ 5**	0.03	0.02	0.98
**75**	**5**	0.07	0.04	0.96
	**6**	0.07	0.04	0.96
	**7**	0.2	0.1	0.9
	**8**	0.25	0.12	0.88
	**9**	0.27	0.14	0.86
	**10**	0.34	0.17	0.83
	**11**	0.27	0.13	0.87
	**12**	0.15	0.08	0.93
	**≥ 13**	0.11	0.05	0.95
	**≥ 5**	0.08	0.04	0.96
**90**	**5**	0.03	0.02	0.98
	**6**	0.03	0.02	0.98
	**7**	0.05	0.03	0.97
	**8**	0.07	0.03	0.97
	**9**	0.22	0.11	0.89
	**10**	0.64	0.32	0.68
	**11**	0.81	0.4	0.6
	**12**	0.42	0.21	0.79
	**≥ 13**	0.44	0.22	0.78
	**≥ 5**	0.03	0.02	0.98

[a]Comparison between short and long-lived subpopulations using the 90th percentile threshold was not performed as the number of long-lived species falls below 10.

Supplementary Table S7: Comparison of Mean DR counts between short lived and long lived mammals

Two-sample t-test was used to test the null hypothesis that DR counts in short-lived and long-lived datasets arise from normal distributions with equal mean values and equal but unknown variances, against the following alternative hypotheses (a) mean DR count values are unequal, (b) mean DR count value in short-lived dataset is higher than the long-lived dataset and (c) mean DR count value in short-lived dataset is lower than the long-lived dataset. Pairwise comparisons with p-values ≤ 0.05 are highlighted in red.

Percentile	DR size	short-lived ≠ long lived	short-lived > long lived	short-lived < long lived
		p-value	p-value	p-value
**Mammals**				
**50**	**5**	0.58	0.29	0.71
	**6**	0.91	0.46	0.54
	**7**	0.66	0.67	0.33
	**8**	0.41	0.80	0.20
	**9**	0.33	0.83	0.17
	**10**	0.11	0.94	0.06
	**11**	0.35	0.82	0.18
	**12**	0.74	0.63	0.37
	**≥ 13**	0.79	0.60	0.40
	**≥ 5**	0.76	0.38	0.62
**75**	**5**	0.73	0.63	0.37
	**6**	0.56	0.72	0.28
	**7**	0.21	0.89	0.11
	**8**	0.08	0.96	0.04
	**9**	0.02	0.99	0.01
	**10**	0.00	1.00	0.00
	**11**	0.00	1.00	0.00
	**12**	0.01	1.00	0.00
	**≥ 13**	0.33	0.84	0.16
	**≥ 5**	0.58	0.71	0.29

**90**	**5**	0.32	0.84	0.16
	**6**	0.19	0.91	0.09
	**7**	0.05	0.98	0.02
	**8**	0.01	1.00	0.00
	**9**	0.00	1.00	0.00
	**10**	0.00	1.00	0.00
	**11**	0.01	1.00	0.00
	**12**	0.09	0.96	0.04
	**≥ 13**	0.80	0.40	0.60
	**≥ 5**	0.21	0.89	0.11
**Primates[a]**		**short-lived ≠ long lived**	**short-lived > long lived**	**short-lived < long lived**
**50**	**5**	0.22	0.89	0.11
	**6**	0.18	0.91	0.09
	**7**	0.09	0.96	0.04
	**8**	0.05	0.97	0.03
	**9**	0.04	0.98	0.02
	**10**	0.01	1.00	0.00
	**11**	0.04	0.98	0.02
	**12**	0.02	0.99	0.01
	**≥ 13**	0.71	0.64	0.36
	**≥ 5**	0.17	0.91	0.09
**75**	**5**	0.65	0.68	0.32
	**6**	0.46	0.77	0.23
	**7**	0.26	0.87	0.13
	**8**	0.18	0.91	0.09
	**9**	0.14	0.93	0.07
	**10**	0.13	0.94	0.06
	**11**	0.65	0.68	0.32
	**12**	0.97	0.48	0.52
	**≥ 13**	0.16	0.08	0.92
	**≥ 5**	0.53	0.74	0.26

**Carnivora**[a]		**short-lived ≠ long lived**	**short-lived > long lived**	**short-lived < long lived**
**50**	**5**	0.34	0.83	0.17
	**6**	0.18	0.91	0.09
	**7**	0.06	0.97	0.03
	**8**	0.03	0.98	0.02
	**9**	0.05	0.97	0.03
	**10**	0.02	0.99	0.01
	**11**	0.06	0.97	0.03
	**12**	0.18	0.91	0.09
	**≥ 13**	0.66	0.33	0.67
	**≥ 5**	0.24	0.88	0.12
**75**	**5**	0.96	0.52	0.48
	**6**	0.76	0.62	0.38
	**7**	0.43	0.79	0.21
	**8**	0.28	0.86	0.14
	**9**	0.27	0.86	0.14
	**10**	0.15	0.93	0.07
	**11**	0.38	0.81	0.19
	**12**	0.48	0.76	0.24
	**≥ 13**	0.71	0.36	0.64
	**≥ 5**	0.83	0.59	0.41
**Artiodactyla**		**short-lived ≠ long lived**	**short-lived > long lived**	**short-lived < long lived**
**50**	**5**	0.01	0.01	0.99
	**6**	0.02	0.01	0.99
	**7**	0.04	0.02	0.98
	**8**	0.06	0.03	0.97
	**9**	0.10	0.05	0.95
	**10**	0.06	0.03	0.97
	**11**	0.02	0.01	0.99
	**12**	0.03	0.02	0.98
	**≥ 13**	0.03	0.01	0.99

	**≥ 5**	0.02	0.01	0.99
**75**	**5**	0.02	0.01	0.99
	**6**	0.02	0.01	0.99
	**7**	0.04	0.02	0.98
	**8**	0.05	0.03	0.97
	**9**	0.10	0.05	0.95
	**10**	0.25	0.12	0.88
	**11**	0.17	0.09	0.91
	**12**	0.07	0.04	0.96
	**≥ 13**	0.30	0.15	0.85
	**≥ 5**	0.02	0.01	0.99
**90**	**5**	0.00	0.00	1.00
	**6**	0.00	0.00	1.00
	**7**	0.00	0.00	1.00
	**8**	0.00	0.00	1.00
	**9**	0.02	0.01	0.99
	**10**	0.35	0.18	0.82
	**11**	0.61	0.30	0.70
	**12**	0.40	0.20	0.80
	**≥ 13**	0.70	0.35	0.65
	**≥ 5**	0.00	0.00	1.00

[a]Comparison between short and long-lived subpopulations using the 90th percentile threshold was not performed as the number of long-lived species falls below 10.

Supplementary Table S8: Comparison of DR count distributions between short-lived and long-lived mammals

Two-sample K-S test was used to test the null hypothesis that DR count distributions in short-lived and long-lived datasets arise from a same distribution against the alternative that they are not. Pairwise comparisons with p-values ≤ 0.05 are highlighted in red.

DR size	Mammals			Primates[a]	
	50th Percentile	75th Percentile	90th Percentile	50th Percentile	75th Percentile
	p-value	p-value	p-value	p-value	p-value
**5 bp**	0.33	0.01	0.00	0.24	0.84
**6 bp**	0.33	0.00	0.00	0.06	0.42
**7 bp**	0.26	0.00	0.00	0.06	0.56
**8 bp**	0.21	0.00	0.00	0.06	0.70
**9 bp**	0.59	0.01	0.00	0.03	0.14
**10 bp**	0.16	0.00	0.00	0.03	0.13
**11 bp**	0.98	0.00	0.00	0.06	0.27
**12 bp**	0.69	0.00	0.03	0.06	0.92
**≥ 13 bp**	0.59	0.98	0.87	0.65	0.38
**≥ 5 bp**	0.41	0.00	0.00	0.24	0.84

DR size	Carnivora[a]		Artiodactyla		
	50th Percentile	75th Percentile	50th Percentile	75th Percentile	90th Percentile
	p-value	p-value	p-value	p-value	p-value
**5 bp**	0.09	0.15	0.12	0.11	0.07
**6 bp**	0.09	0.28	0.12	0.15	0.07
**7 bp**	0.17	0.28	0.28	0.41	0.07
**8 bp**	0.16	0.22	0.19	0.27	0.07
**9 bp**	0.08	0.21	0.54	0.50	0.17
**10 bp**	0.01	0.05	0.21	0.13	0.19
**11 bp**	0.05	0.28	0.13	0.49	1.00
**12 bp**	0.15	0.48	0.36	0.53	0.60
**≥ 13 bp**	0.66	0.94	0.07	0.32	0.58
**≥ 5 bp**	0.09	0.15	0.12	0.15	0.07

[a]Comparison between short and long-lived subpopulations using the 90th percentile threshold was not performed as the number of long-lived species falls below 10.

Supplementary Table S9: Percent reduction in DR counts between human mtDNA and randomized DNA sequences

DR size	Percentage Reduction							
	RGO	USCU	nucleotides reshuffled				codons reshuffled	NU
			rRNA	tRNA	protein	full	protein	
**5**	0.11	28.28	1.60	0.73	8.37	10.93	4.01	41.01
**6**	0.11	34.13	2.13	0.97	12.07	15.46	5.60	48.42
**7**	0.08	40.09	2.79	1.10	17.06	21.30	7.64	55.76
**8**	0.46	45.42	3.43	2.02	21.98	27.19	9.79	62.24
**9**	0.62	48.88	3.07	2.22	24.96	30.89	9.64	66.93
**10**	0.07	49.00	2.19	0.08	22.99	30.06	3.07	69.45
**11**	0.62	38.07	4.21	3.38	5.20	18.19	23.37	67.57
**12**	4.79	26.26	8.05	13.84	19.21	3.58	63.74	63.21
**≥ 13**	5.75	33.00	8.00	19.75	11.13	14.00	56.38	69.88
**≥ 5**	0.12	30.60	1.82	0.83	9.98	12.88	4.66	43.94

Supplementary Table S10: Two-sided comparison of DR counts between human mtDNA and randomized DNA sequences

Two-sided Z-test was used to test the null hypothesis that DR count in native mtDNA arise from a normal distribution with mean and standard deviation DR count values observed in the randomized DNA sequences against the alternative that the mean values are not same. Pairwise comparisons with p-values ≤ 0.05 are highlighted in red.

DR size	Two sided Z-test p-values							
	RGO	USCU	nucleotides reshuffled				codons reshuffled	NU
			rRNA	tRNA	protein	full	protein	
**5**	0.08	0.00	0.00	0.00	0.00	0.00	0.00	0.00
**6**	0.22	0.00	0.00	0.01	0.00	0.00	0.00	0.00
**7**	0.61	0.00	0.00	0.02	0.00	0.00	0.00	0.00
**8**	0.10	0.00	0.00	0.01	0.00	0.00	0.00	0.00
**9**	0.26	0.00	0.05	0.09	0.00	0.00	0.00	0.00
**10**	0.95	0.00	0.44	0.98	0.00	0.00	0.63	0.00
**11**	0.82	0.00	0.46	0.52	0.70	0.18	0.13	0.00
**12**	0.51	0.30	0.57	0.26	0.52	0.91	0.09	0.00
**≥ 13**	0.74	0.71	0.81	0.53	0.88	0.86	0.56	0.03
**≥ 5**	0.11	0.00	0.00	0.00	0.00	0.00	0.00	0.00

Supplementary Table S11: One-sided comparison of DR counts between human mtDNA and randomized DNA sequences

One-sided Z-test was used to test the null hypothesis that DR count in native mtDNA arise from a normal distribution with mean and standard deviation DR count values observed in the randomized DNA sequences against the alternative that the mean DR count values in the randomized sequences are lower than the native mtDNA. Pairwise comparisons with p-values ≤ 0.05 are highlighted in red.

DR size	One sided Z-test p-values							
	RGO	USCU	nucleotides reshuffled				codons reshuffled	NU
			rRNA	tRNA	protein	full	protein	
**5**	0.04	0.00	0.00	0.00	0.00	0.00	0.00	0.00
**6**	0.11	0.00	0.00	0.00	0.00	0.00	0.00	0.00
**7**	0.30	0.00	0.00	0.01	0.00	0.00	0.00	0.00
**8**	0.05	0.00	0.00	0.00	0.00	0.00	0.00	0.00
**9**	0.13	0.00	0.02	0.05	0.00	0.00	0.00	0.00
**10**	0.52	0.00	0.22	0.49	0.00	0.00	0.32	0.00
**11**	0.59	0.00	0.23	0.74	0.35	0.09	0.93	0.00
**12**	0.74	0.15	0.29	0.87	0.74	0.45	0.95	0.00
**≥ 13**	0.63	0.35	0.59	0.74	0.56	0.43	0.72	0.01
**≥ 5**	0.06	0.00	0.00	0.00	0.00	0.00	0.00	0.00

Supplementary Table S12: Normalized Reduction in DR counts between human mtDNA and randomized DNA sequences.

DR size	Normalized Reduction in DR count							
	RGO	USCU	nucleotides reshuffled				codons reshuffled	NU
			rRNA	tRNA	protein	full	protein	
5 bp	0.01	4.92	1.25	0.96	1.46	1.40	0.70	5.24
6 bp	0.00	1.70	0.48	0.36	0.60	0.56	0.28	1.77
7 bp	0.00	0.58	0.18	0.12	0.25	0.23	0.11	0.59
8 bp	0.00	0.19	0.07	0.06	0.09	0.08	0.04	0.19
9 bp	0.00	0.06	0.02	0.02	0.03	0.03	0.01	0.06
10 bp	0.00	0.02	0.00	0.00	0.01	0.01	0.00	0.02
11 bp	0.00	0.00	0.00	0.00	0.00	0.00	0.00	0.00
12 bp	0.00	0.00	0.00	0.00	0.00	0.00	0.00	0.00
≥ 13 bp	0.00	0.00	0.00	0.00	0.00	0.00	0.00	0.00
≥ 5 bp	0.02	7.47	2.00	1.52	2.43	2.31	1.14	7.87

Supplementary Table S13: Correlation between PICs of residual N_c and residual DR counts using ClustalW-Phylip

Correlations with p-values ≤ 0.05 are highlighted in red.

DR size	Correlation between PICs of residual N_c and residual DR count (ClustalW-Phylip)							
	All Mammals		Diprotodontia		Primates		Rodentia	
	ρ	p-value	ρ	p-value	ρ	p-value	ρ	p-value
**5 bp**	-0.32	0.00	0.31	0.35	-0.50	0.00	-0.26	0.30
**6 bp**	-0.30	0.00	0.30	0.37	-0.50	0.00	-0.28	0.26
**7 bp**	-0.30	0.00	0.35	0.29	-0.49	0.00	-0.30	0.22
**8 bp**	-0.26	0.00	0.15	0.66	-0.39	0.01	-0.42	0.08
**9 bp**	-0.23	0.00	0.15	0.67	-0.20	0.16	-0.43	0.07
**10 bp**	-0.24	0.00	0.07	0.83	-0.04	0.81	-0.25	0.32
**11 bp**	-0.23	0.00	0.10	0.76	0.04	0.79	-0.18	0.47
**12 bp**	-0.19	0.00	-0.06	0.86	-0.05	0.71	0.04	0.86
**≥ 13 bp**	-0.13	0.02	-0.24	0.48	0.09	0.55	0.09	0.71
**≥ 5 bp**	-0.32	0.00	0.32	0.34	-0.49	0.00	-0.28	0.25
**DR size**	Carnivora		Artiodactyla		Cetacea		Aves	
	ρ	p-value	ρ	p-value	ρ	p-value	ρ	p-value
**5 bp**	-0.37	0.00	-0.28	0.00	-0.24	0.17	-0.23	0.00
**6 bp**	-0.33	0.01	-0.24	0.01	-0.17	0.33	-0.27	0.00
**7 bp**	-0.34	0.01	-0.26	0.01	-0.12	0.48	-0.24	0.00
**8 bp**	-0.32	0.01	-0.26	0.00	-0.03	0.85	-0.12	0.08
**9 bp**	-0.29	0.02	-0.28	0.00	0.00	1.00	-0.01	0.84
**10 bp**	-0.36	0.00	-0.32	0.00	-0.05	0.77	0.02	0.75
**11 bp**	-0.36	0.01	-0.31	0.00	-0.20	0.26	0.04	0.57
**12 bp**	-0.28	0.03	-0.17	0.07	-0.32	0.07	0.04	0.53
**≥ 13 bp**	-0.27	0.04	-0.13	0.17	-0.30	0.09	0.04	0.50
**≥ 5 bp**	-0.37	0.00	-0.28	0.00	-0.21	0.24	-0.15	0.02

Supplementary Table S14: Correlation between PICs of residual N_c and residual DR counts using MUSCLE-Phylip

Correlations with p-values ≤ 0.05 are highlighted in red.

DR size	Correlation between PICs of residual N_c and residual DR count (MUSCLE-Phylip)							
	All Mammals		Diprotodontia		Primates		Rodentia	
	ρ	p-value	ρ	p-value	ρ	p-value	ρ	p-value
**5 bp**	-0.33	0.00	0.37	0.26	-0.52	0.00	-0.24	0.34
**6 bp**	-0.31	0.00	0.35	0.29	-0.51	0.00	-0.27	0.29
**7 bp**	-0.30	0.00	0.41	0.21	-0.49	0.00	-0.28	0.27
**8 bp**	-0.26	0.00	0.25	0.47	-0.39	0.01	-0.39	0.11
**9 bp**	-0.23	0.00	0.25	0.46	-0.20	0.17	-0.44	0.07
**10 bp**	-0.23	0.00	0.09	0.78	-0.01	0.97	-0.39	0.11
**11 bp**	-0.23	0.00	0.10	0.76	0.08	0.57	-0.36	0.15
**12 bp**	-0.18	0.00	-0.05	0.88	-0.03	0.81	-0.04	0.88
**≥ 13 bp**	-0.12	0.03	-0.10	0.76	0.07	0.65	0.07	0.77
**≥ 5 bp**	-0.33	0.00	0.39	0.24	-0.51	0.00	-0.27	0.29
**DR size**	**Carnivora**		**Artiodactyla**		**Cetacea**		**Aves**	
	ρ	p-value	ρ	p-value	ρ	p-value	ρ	p-value
**5 bp**	-0.35	0.01	-0.29	0.00	-0.21	0.22	-0.24	0.00
**6 bp**	-0.32	0.01	-0.26	0.01	-0.16	0.37	-0.28	0.00
**7 bp**	-0.32	0.01	-0.27	0.00	-0.15	0.41	-0.24	0.00
**8 bp**	-0.29	0.02	-0.27	0.00	-0.06	0.73	-0.11	0.08
**9 bp**	-0.26	0.04	-0.29	0.00	-0.05	0.80	-0.01	0.91
**10 bp**	-0.34	0.01	-0.32	0.00	-0.09	0.62	0.03	0.69
**11 bp**	-0.35	0.01	-0.31	0.00	-0.24	0.18	0.04	0.51
**12 bp**	-0.29	0.03	-0.17	0.06	-0.34	0.05	0.05	0.47
**≥ 13 bp**	-0.27	0.04	-0.14	0.14	-0.35	0.04	0.05	0.43
**≥ 5 bp**	-0.36	0.01	-0.29	0.00	-0.19	0.27	-0.16	0.02
