# Supplementary material for: Are mutagenic non D-loop direct repeat motifs in mitochondrial DNA under a negative selection pressure?
Source: Nucleic Acids Res. 2015 Apr 8;43(8):4098–108. doi: 10.1093/nar/gkv299 (PMC4417187; doi:10.1093/nar/gkv299)
Supplement: SUPPLEMENTARY DATA [file supp_gkv299_nar-02864-z-2014-File009.pdf]

Supplementary Figures

Table of Contents

Supplementary Figure S1: Extent of bias in synonymous codon usage in mammalian mtDNA sequences.......................... 2

Supplementary Figure S2: Correlation between mtDNA SCU bias and total mtDNA DR frequency.. 3

Supplementary Figure S3: N_c-plot for individual taxonomic orders 4

Supplementary Figure S1: Extent of bias in synonymous codon usage in mammalian mtDNA sequences.

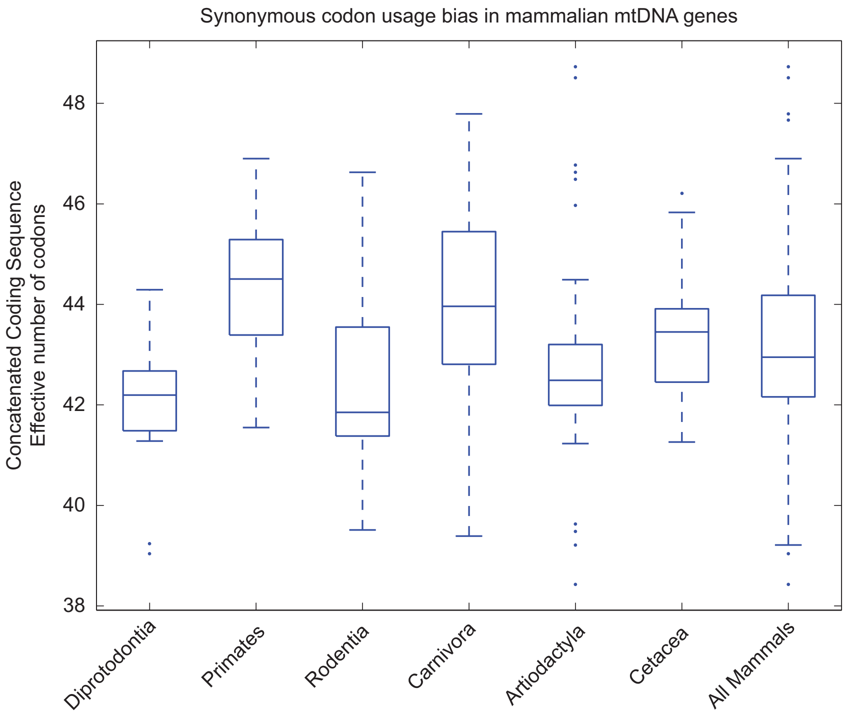

Distribution of N_c values of concatenated mtDNA protein coding genes in individual taxonomic orders in mammals.

Supplementary Figure S2: Correlation between mtDNA SCU bias and total mtDNA DR frequency

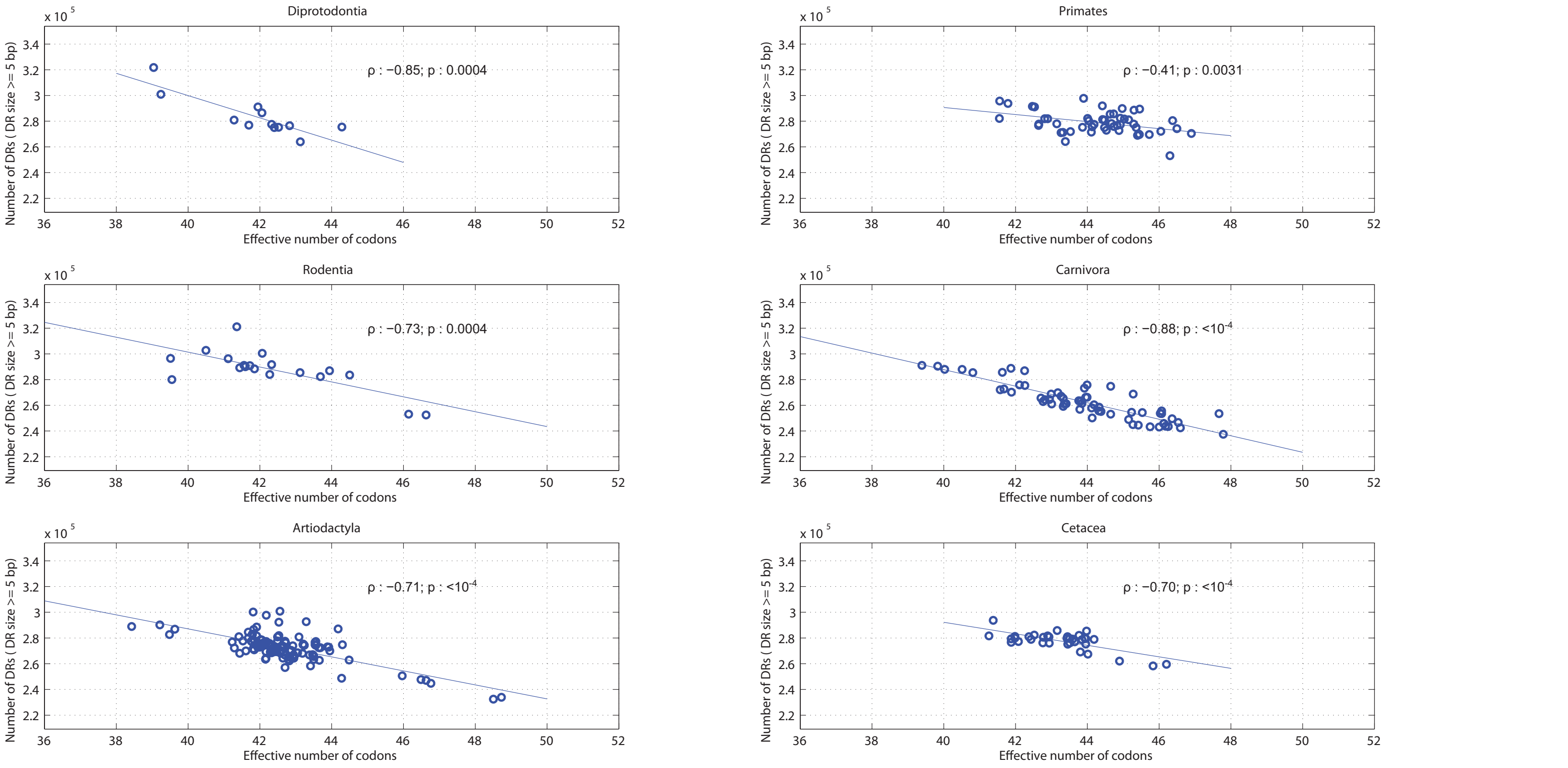

Correlation analysis in taxonomic orders within mammals. There exists significant negative correlation between the calculated effective number of codons and the total number of DRs in all the data sets. The linear correlation coefficient (r) is shown for each plot.

Supplementary Figure S3: N_c-plot for individual taxonomic orders

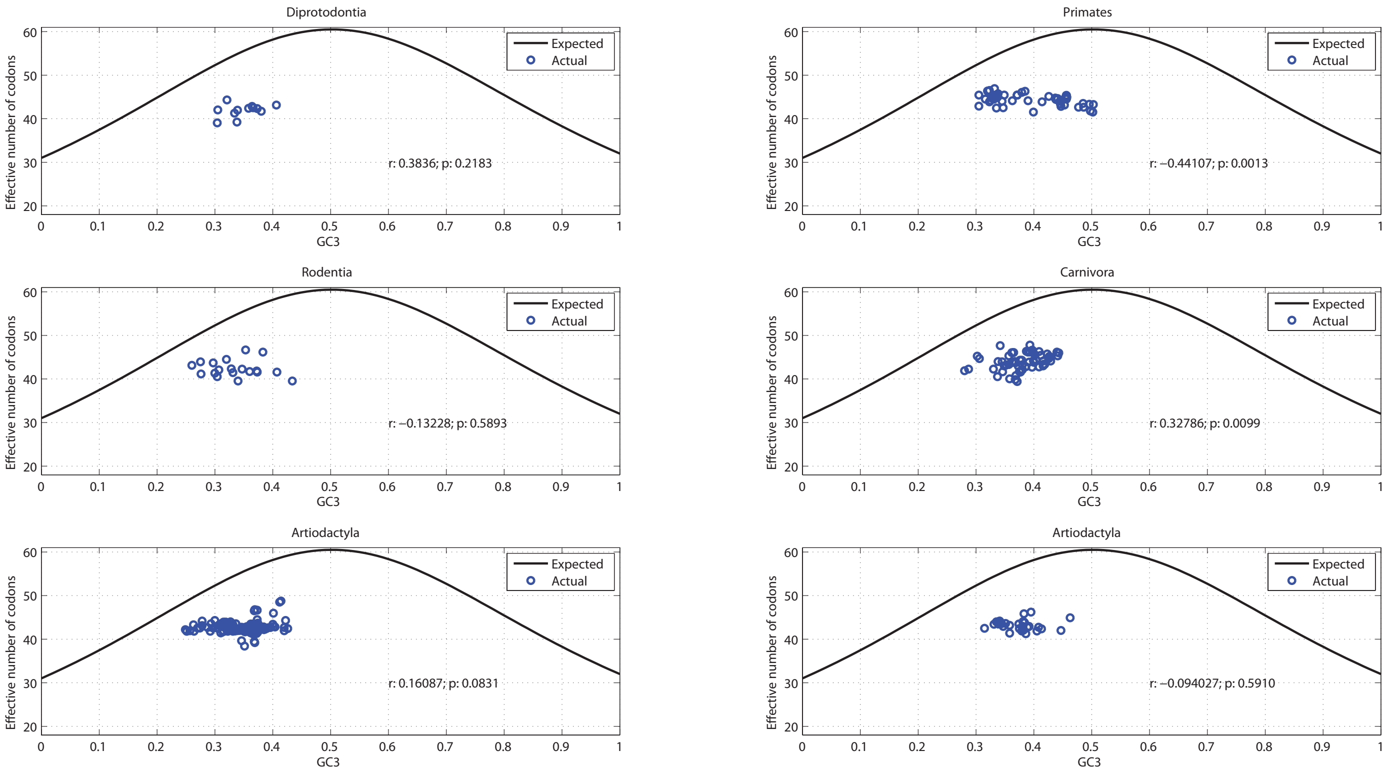

Relationship between the expected SCU bias due to only nucleotide mutational bias (N_c^*; in black solid line), the SCU bias (effective number of codons) in individual taxonomic orders within the mammalian mtDNA sequences compendium (N_c; in blue circles) and the GC fractions at third codon positions. The correlation coefficient (ρ) and the p-value for the correlation between the calculated effective number of codons and the GC3 are mentioned within each plot.
